# Bioprocessing strategies for cost-effective large-scale biogenic synthesis of nano-MgO from endophytic *Streptomyces coelicolor* strain E72 as an anti-multidrug-resistant pathogens agent

**DOI:** 10.1038/s41598-018-22134-x

**Published:** 2018-02-28

**Authors:** Shahira H. EL-Moslamy

**Affiliations:** Department of Bioprocess development, Genetic Engineering and Biotechnology Research Institute, City of Scientific Research and Technology Applications, New Borg El-Arab City, Alexandria, Egypt

## Abstract

In this report, the local nano-MgO synthesizer strain has been isolated from *Ocimum sanctum* plant and deposited in GenBank as endophytic *Streptomyces coelicolor* strain E72. Its intracellular metabolic fraction that contains 7.2 μg/μl of carbohydrate, 6.3 g/l of protein and 5.2 nmol/hr/ml of nitrate reductase used to produce multi-surface shaped nano-MgO with diameter ~25 nm. To the best of our knowledge, this is the first report using statistical nanobiotechnological strategies (Plackett -Burman, Box-Behnken and Taguchi experimental designs) to study and evaluate the endophytic *S*. *coelicolor* biomass production (123.3 g/l) and extract the highest bioactive metabolites that used for biogenic synthesis of nano-MgO (320 g/l) through exponential sucrose pulses feeding fermentation strategy after 192 hr in semi industrial scale bioreactor (7 L). Purified nano-MgO applied *in vitro* against multi-drug resistant human pathogens and the large inhibition zone recorded against *Shigella flexneri* (108 ± 10.53 mm). The average of MICs was recorded as 25 µg/ml that inhibited 90% of the pathogenic living cells and compared with 100 mg/ml ampicilin/sulbactam solution that killed 40% of the same pathogen. These results are expected to gather sufficient knowledge to discover and develop a new cheap and eco-friendly nano-MgO as an extremely strong antimicrobial agent used in biomedical applications.

## Introduction

There are different human pathogenic species resist all known antibiotic classes and organic antimicrobial agents (bacteriocins and enzymes, etc), so the pharmaceutical companies and the researchers planned to emerge up from this problem by discovering and developing other antimicrobial agents^1–4^. Synthesized nanoparticles recognized as effective antimicrobial agents used in different biomedical applications due to their morphological structure, stability, and surface properties^5–10^. In recent years, the most studied nano-science publications interested in bioprocessing development for metal oxide nanoparticles that used as novel antimicrobial agents due to their ability to fight and control multi-drugs resistant microbial infections^2,3,5,6^. The explored nanoparticles that significantly effect on the growth of different human pathogenic species can be listed in the following sequence Ag > Hg > Cu > Cd > Cr > Pb > Co > Au > Zn > Fe > Mn > Mo > Sn^11–17^. Magnesium oxide nanoparticles are an attractive metal oxide material that used for bio-medical, agriculture and electrochemical applications^7–9^. Recently, the potential bio-applications of the bio-synthesized nano-MgO such as a curative catalyst agent, anticancer drug, anti-diabetic agent, anti-microbial agent, electrochemical biosensor, remediation agent, paints and superconductors were reported in several studies^5–10,14–17^. Nano-MgO was applied also as antimicrobial agent that induced the systemic resistance in plant against microbial wilt disease that caused by phytopathogenic bacteria and/or phytopathogenic fungi^18–20^. According to the cytotoxic profiles, there are many reports strongly highlight that the low concentration of the bio-synthesized nano-MgO are less toxic nanoparticles to human cells with time then can be applied safely in the therapeutic, agriculture and water treatment applications with lass potential hazards to the environment and humans^21–23^. Nano-MgO has significant characters such as long-term thermal stability at high levels, low electrical conductivity, and low toxicity compared with nano-Ag that draw the attention of many scientists on the development of those nanoparticles at industrial level by using the safe bio-processing strategy^18–22^. Nano-MgO can be synthesized conventionally by using chemical or physical techniques via sol-gel, hydrothermal, flame spray pyrolysis, laser vaporization, chemical gas phase deposition, combustion aerosol synthesis, aqueous wet chemical, and surfactant methods^21–23^. Nano-MgO that synthesized by using these complicated and expensive techniques produced hazardous residues which affected on its bio-application for biological and medical uses^13–17^. So, biogenic methods served as proficient alternate methods to produce nontoxic nano-MgO for biological and therapeutic applications by using plant extracts, algae, bacteria, fungi and yeasts^16–19^. Since the extracted safe bioactive metabolites (bio-chemicals, enzymes, vitamins, alkaloids, phenols, proteins, and polysaccharides) can be used as reducing/capping agents and/or stabilizing agents for the biogenic synthesis of nano-MgO^20,21^. So the fabrication of nano-MgO by using biogenic techniques has different and significant characters due to their simplicity, environmental sustainability, cost-effectiveness, reproducibility compared with physical and chemical techniques^22,23^. The most important factor that affecting on the biosynthesis of nanoparticles which used in biomedical applications is nanoparticles synthesizer because it should be safe and produce the highest bio-reluctant concentration by using low cost of cultivation method^23–25^. This factor followed by others such as reaction period, temperature, pH, and its nano-form stability. Due to all the previous characters *actinomycetes* are attractive safe microorganism used for biogenic synthesis of nanoparticles. Nowadays, there are many advanced nano-biotechnological strategies, have emerged can be used as tools to improve the production of natural bioactive metabolites and their activities to reduce the production line cost. Bad construction of the optimization technique that depends on the effect of one variable at a time leads to misinterpretation of the final results because of the omission of interaction effect among different variables such as medium parameters, biosynthesis of nanoparticles factors and so on^23–25^. So, statistical experimental designs are ideal promising tools that recently extensively applied and reported as proficient and effective methodologies such as Plackett - Burman design (PBD), Box - Behnken design (BBD) and Taguchi design methods (TD) that evaluate the desired natural products which used for different industrial applications as much as possible^23–25^. These designs depended on specific sequence steps such as screening for the most effective factors that selected for studying the interaction effect among them and calculating their optimum concentrations which affect on desirable responses^23–25^.

Medicinal plants are precious safe sources of novel microbial and potent bioactive compounds diversity that used as new drug candidates (raw materials). It is important to note that these treasure troves of natural drugs are most effective with negligible side effects and investment costless available^26–28^. Endophyte is a microbe that colonizes in the intracellular or intercellular tissues of healthy plant and explored as an important part of micro-ecosystem^29–31^. Endophytic microbe’s flora that isolated from these medicinal plants has the same and/or similar bioactive metabolites, thus making them a proficient and unique source of novel safe natural compounds for medicinal applications^32–35^. Several studies have indicated that endophytes are distributed in roots, stems, leaves, flowers, fruits and seeds which classified into endophytic bacteria, actinomycetes and fungi^36–38^. In recent years, secondary metabolites of isolated endophytes used as antitumor, antimicrobial, antioxidant, and other functions that have a potential application prospect in pharmacy^28,31,36–38^. Until now, very few researchers have reported endophytic actinomycetes as nanoparticles biosynthesizer by using nanobiotechnological strategies^29,30^. So, endophytic actinomycetes have attracted attention in the search for an increasingly important source of promising potential new bioactive compounds that used as novel nanoparticles-synthesizers^26–28,34,35^. To the best of our knowledge, there are no reports on the biosynthesis of nano-MgO using an endophytic *Streptomyces* sp. So this study focuses on isolation of endophytic *Streptomyces sp* from *Ocimum sanctum* and using promising statistical techniques for *Streptomyces sp* cultivation and biogenic synthesis of nano-MgO that was characterized and its antimicrobial activity was evaluated against clinical multi drug resistant strains.

## Methods

### Collection of the plant, surface sterilization and *Actinomycetes* isolation

Healthy plants of *Ocimum sanctum* were randomly selected from Lower Egypt herbal gardens. Samples were placed in sterile polythene bag cover and brought to the laboratory in boxes. A Leaf sample was washed with tap water for many times to remove adhered soil debris and with sterilized water for three times in sterile beaker. After that the leaf sample was surface-sterilized with 70% ethyl alcohol for 5 min and subsequently with absolute ethyl alcohol for 2 min^32,36^. The ethanol sterilized leaves were dried on sterilized filter paper for 10 min and then were treated with 6% sodium hypochlorite with Tween 20 (0.1%) for 2 min to remove epiphytic microorganisms. The leaves were soaked in 10% NaHCO_3_ for 20 min to disrupt the growth of the fungi^29,31^. At last leaves rinsed with sterilized distilled water for many times and sterility checked (the final rinsing water was spread on starch casein agar as the control plates)^23–25^. The surface sterilized leaves were cut with sterile scalpel blade and crushed in sterilized distilled water by using sterile pestle and mortar. By using serial dilution method 100 μl of the diluted extract was spread on starch casein agar (0.1% sodium caseinate, 1% soluble starch, 0.03% K_2_HPO_4_, 1.8% agar, pH 6.8–7.0) supplemented with 25 µg/ml cycloheximide, 50 μg/ml nalidixic acid and 50 μg/ml nystatin to suppress fungal growth and incubated at 30 °C for three weeks^29,31^. *Actinomycetes* growing on the media were isolated and purified. Purified isolates were stored at −80 °C in 50% (v/v) glycerol for long term use. Finally, the pure cultures can be inoculated on the broth medium for bioprocessing fermentation studies.

### Screening of isolated *Actinomycetes* for nano-MgO biosynthesis

Each isolate was grown in 100 ml of MGYP medium (0.3% malt extract, 1% glucose, 0.3% yeast extract, 0.5% peptone and the final pH was 6.8) at 30 °C for 72 hr. Balls of mycelia were separated by centrifugation (10,000 rpm) at 4 °C for 15 min and then the mycelia were washed thrice with sterile distilled water. Wet weight of the harvested biomass (20 g) was re-suspended in 100 ml sterile distilled water and incubated on a shaker (150 rpm) at 30 °C for 72 hr; then the cell-free filtrate was obtained by using ultrafiltration system. Biosynthesis reaction for nano-MgO was set up by a mix of 90 ml of precursor metal salt (1 M Mg(NO_3_)_2_·6H_2_O) with 10 ml of extracellular bioactive compounds (pH 5.0) at 80 °C under constant mechanically stirring for 2 hr and allowed to settle for 24 hr^9,39^. The upper layer was discarded carefully and the remaining pellet was centrifuged at 14,000 rpm for 25 min. This pellet was washed several times to remove the impurities^5,8^. Subsequently, the separated pellet was dried in an oven at 80 °C for 12 hr and then crushed to make it very fine powder by using a mortar pestle. Finally, the fine powder of nano-MgO is calcinated at 700 °C for 3 hr for the removal of impurities. Nano-MgO antibacterial activity was screened against Gram−ve and Gram + ve bacteria (*Escherichia coli*, *Proteus vulgaris*, *Salmonella typhimurium*, *Shigella flexneri*, *Klebsiella pneumonia*, *Staphylococcus aureus*, *Streptococcus pneumonia* and *Bacillus cereus*) by using agar well diffusion method and compared with standard conventional antibiotics by using disc diffusion method^2,4,7,40,41^.

### Physicochemical properties of prepared nano-MgO

The initial characterization for prepared nano-MgO was monitored by measuring the UV–vis absorbance spectrum by using a UV-Visible Spectroscopy (Shimadzu, Tokyo, Japan) to detect the surface plasmon resonance band^6,8–10^. The structural properties (real estate properties) of the nano-MgO were analyzed by transmission electron microscope (JEOL JEM2100F- Japan), scanning electronmicroscopy (SEM) (JEOL JSM 6360LA, Japan), scientific particle size analyzer (PSA) and its chemical composition was demonstrated by an energy dispersive X-Ray (EDX) analyzer. The FTIR spectrums were measured using Shimadzu FTIR-8400 S, Japan, over the wavelength range 400–4000 cm^−1^. X-ray diffraction patterns were obtained using Schimadzu 7000 diffractometer operating with Cu Kα_1_ radiation(λ = 0.15406 nm) generated at 30 kV and 30 mA with the scan rate of 2°/min for 2θ values between 20° and 80°. The average particle size was estimated using the Scherrer formula Eq. (1).

1 $${\bf{D}}=k{\boldsymbol{\lambda }}/{\boldsymbol{\beta }}\,\cos \,{\boldsymbol{\theta }}$$

where **D** is particle diameter, **k** is a constant equals 0.9, **λ** is wavelength of X-ray source (0.1541 nm), **β** is the full width at half maximum (FWHM) and θ is the half diffraction angle.

### Molecular identification of the endophytic *Actinomycetes* isolate

Genomic DNA was extracted by using spin-column DNeasy Blood & Tissue Kit according to the QIAGEN quick- prep method. The 16 S ribosomal RNA gene was amplified by using the PCR reaction mixture (25 μl) that contained 80 ng of template DNA, 10 mM each dNTP, 1.5 mM MgCl_2_, 10pmol of each primer (5′ AGT TTG ATC CTG GCT CAG 3′ and 5′ ACG GCT ACC TTG TTA CGA CTT 3′), and 0.5 μl of 500 U Taq DNA polymerase. PCR amplification program was carried out for 40 cycles of 95 °C for 1 min (denaturing), 52 °C for 1 min (annealing) and 72 °C for 1 min (extension). The initial denaturing temperature was 95 °C extended to 5 min; the final extension was for 10 min at 72 °C and then cooled to 4 °C^3,4,15,16,42^. The purified PCR product was sequenced directly according to the protocol recommended by the manufacturer (Model 3130 automated DNA sequencer, Genetic Analyzer, Applied Biosystems, Hitachi, Japan). The sequence was compared for similarity with the homologous sequences using NCBI BLAST available at http://www.ncbinlmnih.gov/. Multiple sequence alignment was carried out by using BioEdit software (Hall, 1999), and the phylogenetic tree was constructed using the neighbor-joining (NJ) method through an MEGA6 program.

### Screening of the selected endophytic *Actinomycetes* bioactive metabolites for biogenic synthesis of nano-MgO

To measure the effectiveness broth culture media that used for the highest biomass production and hence the maximum bioactive metabolites production which used as reducing/stabilizing agent for biogenic synthesis of nano-MgO the comparative evaluation strategy for cultivation media was performed in this study. The compositions of the broth culture media were selected from the previous publications^43–48^. Modified Czapek-Dox medium **(M1)** consisted of (g/l): Sucrose, 30.0 g; (NH_4_)_2_SO_4_, 2.0 g; NaNO_3_, 1.0 g; K_2_HPO_4_, 1.0 g; KCl, 0.5 g; MgSO_4_·7H_2_O 0.5 g and FeSO_4_7H_2_O, 0.01 g, Yeast Malt Broth **(M2):** malt extract, 6.0 g; maltose, 1.8 g; dextrose, 6.0 g; and yeast extract, 1.2 g, Starch casein agar **(M3):** Soluble starch, 10.0 g; casein, 0.3 g; KNO_3_, 2.0 g; NaCl, 2.0 g; K_2_HPO_4_, 2.0 g; MgSO_4_·7H_2_O, 0.05 g; CaCO_3_, 0.02 g; FeSO_4_·7H_2_O, 0.01 g; pH 7.0, Basal synthetic medium **(M4)**: (NH_4_) H_2_PO_4_, 2.0 g; Dextrose, 20.0 g; K_2_HPO_4_, 1.0 g; MgSO_4_·7H_2_O, 0.5 g; CaCl_2_·2H_2_O, 0.04 g; FeSO_4_·7H_2_O, 0.005 g, and ZnSO_4_·7H_2_O, 0.0005 g, and finally, Glucose Soyabean meal broth **(M5)** that containing: Glucose, 10.0 g; Soyabean meal, 10.0 g; NaCl, 10.0 g; CaCO_3,_ 1.0 g and pH adjusted to 7.0. The cultivation was carried out in 250 ml shake flasks containing 100 ml of the different media separately that sterilized at 121 °C for 15 min and inoculated by 5discs of vegetative cells. The cultivation temperature was controlled at 26 ± 2 °C for 3 days with shaking in an orbital rotary shaker (150 rpm). The biomass was harvested through ultrafiltration system and washed several times with distilled water in order to remove any impurities. By using a clean spatula the pure biomass was transferred to a pre-weighed dry Whatman filter paper and then placed in an oven at 50 °C to reach a fixed weight which expressed as g/l of culture medium^23–25^.

High functional bioactive compounds yielding through cell fractionations were checked (extracellularly and intracellularly). After fermentation step, the broth culture was centrifuged at 14,000 rpm for 20 min to separate the supernatant and the pellet^1,25,43,49^. To extract the extracellular metabolites equal volume of ethyl acetate was added to the supernatant and the mixture was kept for overnight in a rotary shaker. The filtrates were then evaporated using a rotary evaporator at 40 °C. The crude intracellular bioactive metabolite was extracted from the washed biomass (the pellet) that treated with CTAB extraction buffer (100 mM Tris-Cl (pH 8.0), 20 mM EDTA (pH 8.0), 1.4 M NaCl, 2% (w/v) cetyltrimethyl ammonium bromide and 1% PVP 40,000 (polyvinyl pyrrolidone) for 2 hr under shaking condition and finally centrifuged at 14000 rpm for 10 min^44,50,51^. The concentrated bioactive metabolites extracts were then transferred into clean glass screw cap tube and stored at 4 °C for further use.

Total contained proteins in both concentrated cell fractionations were quantified by using the commercial Bio-Rad Colorimetric Protein Assays kit. Also, the total carbohydrate contents of both cell fractionations were determined by using the total carbohydrate colorimetric assay kit (Milpitas, CA 95035 USA). The nitrate reductase enzyme in both cell fractionations was checked and assayed according to^29,31,52^. Cell fractionations were mixed with 10 ml of assay medium (30 mM KNO_3_ and 5% isopropanol in 0.1 M phosphate buffer of pH 7.5) and incubated at 30 °C for 1 hr in the dark condition. After incubation, nitrites formed into the assay mixture (pink color) after adding 1 ml of sulphanilamide and NEED (N-(1-naphthyl) ethylenediamine dihydrochloride) solutions^32,38^. The enzyme activity was estimated by using an UV–vis spectrophotometer at 440 nm and finally expressed in terms of nM of nitrite/hr/ml. Finally, nano-MgO biosynthesis reaction was set up by a mix of 90 ml of precursor metal salt (1 M Mg(NO_3_)_2_·6H_2_O) with 10 ml of both cell fractionations (pH 5.0) at 80 °C under constant mechanically stirring for 2 hr and allowed to settle for 24 hr^9,14,17,53,54^. The centrifuged pellet (at 14,000 rpm for 25 min) was washed several times dried in an oven at 80 °C for 12 hr. The fine powders of nano-MgO weight are estimated (g/l) at these comparative studies.

### Cost-effective optimization strategies for high biomass production

The traditional non-statistical optimization strategies, one-factor-at-a-time approach (OFAT), Is incompetent to detect the true optimum due to the complex interactions among various used parameters^23–25,41,55–57^. Recently, statistical experimental design methodologies considered as powerful tools used for the optimization of the target bioactive metabolite production. In order to it has overcome many obstacles; therefore it can solve multivariate problems to check the significance of several variables and their complex interactions. So statistical experimental designs such as Plackett-Burman design (PBD) and Box-Behnken design (BBD) were applied in this report to produce the highest cost effectiveness biomass production and reduce the fermentation processing time. SigmaPlot 12.5 and Minitab 15.0 were used for these experimental designs and subsequent analysis of the final experimental data.

The statistical optimization strategies for biomass production were carried out in two steps by using fractional factorial method (PBD) followed by response surface method (BBD). Fractional factorial method (PBD) was adopted for screening the most significant nutritional parameters for biomass and bioactive metabolites production by using identified *Streptomyces* strain in submerged fermentation. In the experimental design, seven independent variables (Sucrose, (NH_4_)_2_SO_4_, NaNO_3_, KCl, K_2_HPO_4_, MgSO_4_·7H_2_O, and FeSO_4_·7H_2_O) were screened to pick significant factors that influence biomass production by representing them at two levels (high and low) in eight experiments. All trials were set up in 250 ml Erlenmeyer flasks containing 100 ml of the selected medium (basal medium) and carried out in triplicate to calculate the average biomass weight (the response). The significance of variables was determined by calculating the *p-*value (<5%) and confidence levels through standard regression analysis^23–25,55^. The quality of the first order polynomial model equation was applied statistically by the coefficient of determination R^2^, and its statistical significance was tested by an F-test. Finally, the significant variables were selected for further optimization study. The 1^st^ order model and main effect the response represented in Eqs (2) and (3).

2 $${\boldsymbol{Y}}={{\boldsymbol{\beta }}}_{{\boldsymbol{o}}}+{\boldsymbol{\Sigma }}{{\boldsymbol{\beta }}}_{{\boldsymbol{i}}}{{\boldsymbol{x}}}_{{\boldsymbol{i}}}$$

where **Y** is the response (biomass production), **β**_**0**_ is the models intercept, **β**_**i**_ is the variable estimate, and **X**_**i**_ represents the variable.

3 $${\bf{M}}{\bf{a}}{\bf{i}}{\bf{n}}\,{\bf{e}}{\bf{f}}{\bf{f}}{\bf{e}}{\bf{c}}{\bf{t}}=\sum \,(+{\bf{1}})/{\bf{n}}\,(+{\bf{1}})\,-\sum \,(-{\bf{1}})/{\bf{n}}\,(-{\bf{1}})$$

A 3-level for 3 factors BBD is a powerful most common statistical tool that widely employed for the optimization of industrial fermentation bioprocesses by investigating the interactive effect of the tested variables on product yield through a mathematical model^23–25,41,55^. In this report, the BBD consisted of fifteen trials and the three independent variables (Sucrose, (NH_4_)_2_SO_4_ and K_2_HPO_4_) were studied at three different levels, (−1), (0), (+1), and the dependent variable (response) was biomass production to determine their optimum levels for maximum biomass production. Statistical analysis of this model was performed to evaluate the analysis of variance (ANOVA). The created model was applied using the coefficient results of each variable. The second-order polynomial structured represented in Eq. (4).

4 $${\bf{Y}}={{\boldsymbol{\beta }}}_{{\bf{0}}}{\boldsymbol{+}}{{\boldsymbol{\beta }}}_{{\bf{1}}}{{\bf{X}}}_{{\bf{1}}}{\boldsymbol{+}}{{\boldsymbol{\beta }}}_{{\bf{2}}}{{\bf{X}}}_{{\bf{2}}}{\boldsymbol{+}}{{\boldsymbol{\beta }}}_{{\bf{3}}}{{\bf{X}}}_{{\bf{3}}}{\boldsymbol{+}}{{\boldsymbol{\beta }}}_{{\bf{11}}}{{{\bf{X}}}_{{\bf{1}}}}^{{\bf{2}}}{\boldsymbol{+}}{{\boldsymbol{\beta }}}_{{\bf{22}}}{{{\bf{X}}}_{{\bf{2}}}}^{{\bf{2}}}{\boldsymbol{+}}{{\boldsymbol{\beta }}}_{{\bf{33}}}{{{\bf{X}}}_{{\bf{3}}}}^{{\bf{3}}}{\boldsymbol{+}}{{\boldsymbol{\beta }}}_{{\bf{12}}}{{\bf{X}}}_{{\bf{1}}}{{\bf{X}}}_{{\bf{2}}}{\boldsymbol{+}}{{\boldsymbol{\beta }}}_{{\bf{13}}}{{\bf{X}}}_{{\bf{1}}}{{\bf{X}}}_{{\bf{3}}}{\boldsymbol{+}}{{\boldsymbol{\beta }}}_{{\bf{23}}}{{\bf{X}}}_{{\bf{2}}}{{\bf{X}}}_{{\bf{3}}}$$

where: **Y** is the response (biomass production); **β**_0_ is the model intercept; **X**_**1**_, **X**_**2**_ and **X**_**3**_ are independent variables; **β**_**1**_, **β**_**2**_ and **β**_**3**_ are linear coefficients; **β**_**12**_, **β**_**13**_ and **β**_**23**_ are cross product coefficients; and **β**_**11**_, **β**_**22**_ and **β**_**33**_ are the quadratic coefficients. The confidence level and the accuracy of the model have been calculated by using Eqs (5)and (6).

5 $${\bf{T}}{\bf{h}}{\bf{e}}\,{\bf{c}}{\bf{o}}{\bf{n}}{\bf{f}}{\bf{i}}{\bf{d}}{\bf{e}}{\bf{n}}{\bf{c}}{\bf{e}}\,{\bf{l}}{\bf{e}}{\bf{v}}{\bf{e}}{\bf{l}} \% =100\,\ast \,(1-{\boldsymbol{P}}\,{\bf{v}}{\bf{a}}{\bf{l}}{\bf{u}}{\bf{e}})$$

6 $${\bf{A}}{\bf{c}}{\bf{c}}{\bf{u}}{\bf{r}}{\bf{a}}{\bf{c}}{\bf{y}}\,{\bf{o}}{\bf{f}}\,{\bf{t}}{\bf{h}}{\bf{e}}\,{\bf{m}}{\bf{o}}{\bf{d}}{\bf{e}}{\bf{l}}\,=\,{{\bf{Y}}}_{{\bf{E}}{\bf{x}}{\bf{p}}{\bf{e}}{\bf{r}}{\bf{i}}{\bf{m}}{\bf{e}}{\bf{n}}{\bf{t}}}/{{\bf{Y}}}_{{\bf{C}}{\bf{a}}{\bf{l}}{\bf{c}}{\bf{u}}{\bf{l}}{\bf{a}}{\bf{t}}{\bf{e}}{\bf{d}}}\,\times \,100$$

The simultaneous effects of the three most significant independent factors on each response were visualized using three-dimensional graphs generated by stat soft Sigma plot 12.5 soft ware. The value of each X_1_, X_2_ and X_3_ was further optimized to calculate the highest Y value using Microsoft excel 2007 solver. The experiments for calculating optimum value of each variable were then conducted. All experiments were carried out in triplicates and finally, the mean values were represented.

### Scaling up fermentation strategies

In an accurate industrial bioprocesses strategy, living microbial cells used as cell factories that produce bioactive metabolites by utilizing mathematical kinetic models which are able to develop these cell properties and/or the final production bioprocesses^23–25,46,51,55^. Kinetic cell factory modeling used to understand, predict, develop and assess the effects of modifying (adding and/or removing) cell cultivation components and/or cultivation conditions^23–25,44^. In this work, scaling up strategies performed by using semi-uncontrolled batch, controlled batch and exponential pulses fed-batch cultivation systems. From the previous steps the optimized medium were used for large scale production system. The main objective of this experiment was to set up the large scale fermentation system and estimate the microbial growth kinetics in a submerged cultivation system.

#### Shake-flask cultivation system

The prepared seed culture was conducted by inoculating a 250 ml Erlenmeyer flask containing 50 ml of the optimized medium with a spore suspension (prepared from a 15-day starch nitrate agar culture) and incubating at 30 °C with constant shaking (150 rpm) for 120 hr and then re-inoculated into 1 L flasks containing 500 ml of the optimized medium and cultured at the same conditions. Samples were periodically removed, centrifuged at 10,000 rpm for 15 min (at 4 °C) for measurements of biomass dry weight and for the preparation of intracellular fractions for biosynthesis of nano-MgO as described in the “Screening of isolated *Actinomycetes* for nano-MgO biosynthesis” section.

#### Stirred-bioreactor batch cultivation system

Batch fermentations were conducted in a 7 L Bioflo 310 fermentor (New Brunswick Scientific, Edison, NJ, USA) at 30 °C under controlled pH, temperature and DO concentration. The bioreactor vessel that contained the statistically optimized medium was sterilized by autoclaving at 121 °C for 25 min, while sucrose solution was sterilized separately by autoclaving at 121 °C for 15 min and added aseptically to the bioreactor and then inoculated with prepared inoculums 10% (v/v). The fermentation temperature was adjusted at 30 °C and the agitation speed and aeration rate started at 150 rpm and 0.5 vvm respectively and the dissolved oxygen concentration was maintained at above 40% of air saturation by changing the agitation speed and by regulating the air supply. The pH was controlled at 6.8 by adding 5 N NaOH and/or 5 N HCl. Antifoam (silicone oil 0.5:10 v/v) at a concentration of 1:100 (v/v) in distilled water was added after 24 hr. Samples (50 ml each) were collected periodically and analyzed for biomass, sugar consumption, and nano-MgO production as described previously.

#### Stirred-bioreactor fed-batch cultivation system

The fed batch experiment was designed for carbon source feeding to increase the bioactive metabolites by overcoming carbon limitations. Fed-batch cultivation strategy was developed and conducted with sucrose exponential pulses feeding system by using the optimized batch cultivation results. Feeding was carried out between 96 and 255 hr with the rate of 0.84 g/hr using a prepared sucrose solution (700 g/l) and keeping the dissolved oxygen (DO) value at 40% saturation. Finally, biomass dry weight, sugar consumption, and nano-MgO production were analyzed as described previously. The behavior of microbial cell growth was described kinetically by using different fermentation mode equations^23–25,44,59^. Yield coefficient was calculated based on the amount of sucrose consumed and the growth yield during cultivation. In the log phase, cell mass density increases exponentially with time *t* and specific growth rate *μ* (h^−1^) is independent of nutrient concentration. Since the growth kinetic relationship is affected by many parameters such as biomass yield coefficient (***Y***_*X/S*_), Maximum biomass (**X**_**max**_), Maximum nano-MgO production (**P**_**max**_), nano-MgO production yield coefficient (**Y**_**P**_) and maximum specific growth rate (**µ**_**max**_). Yield coefficient was calculated by using Eq. (7).

7 $${{\boldsymbol{Y}}}_{{\boldsymbol{X}}/{\boldsymbol{S}}}=\frac{{\rm{\Delta }}{\boldsymbol{X}}}{{\rm{\Delta }}{\boldsymbol{S}}}=\frac{{\boldsymbol{X}}-{{\bf{X}}}_{0}}{{{\bf{S}}}_{0}-{\boldsymbol{S}}}$$

where: ***Y***_***X/S***_ Biomass yield on substrate, **X** Cell concentration, **X**_**0**_ Initial cell concentrations, **S** Substrate, **S**_**0**_ Initial substrate concentration. **X** and **X**_**0**_ are biomass concentrations (g/l) at measuring time **t** and initial time **t**_**0**_ respectively. **S** and **S**_**0**_ are the consumed amounts of carbon source (g/l) at the same times mentioned previously. In logarithmic growth phase, cell biomass density increases exponentially with time **t** and specific growth rate **μ (h**^−1^**)** is independent of nutrient concentration as shown in Eq. (8). The most common kinetic model for cell growth was used to determine the maximum specific cell growth rate (Eq. 9). Finally, If the specific growth rate **μ** is constant; the feeding rate calculated by using Eq. (10).

8 $$\frac{{{\boldsymbol{d}}}_{{\boldsymbol{x}}}}{{{\boldsymbol{d}}}_{{\boldsymbol{t}}}}={\boldsymbol{\mu }}X,{\boldsymbol{X}}={{\boldsymbol{X}}}_{0}\,{\boldsymbol{at}}\,{\boldsymbol{t}}=0\to {\bf{l}}{\bf{n}}(\frac{{\boldsymbol{X}}}{{{\boldsymbol{X}}}_{0}})={\boldsymbol{\mu }}t,{\boldsymbol{or}}\,{\boldsymbol{X}}\,=\,{{\boldsymbol{X}}}_{0}\,{{\boldsymbol{e}}}^{{\boldsymbol{\mu }}t}$$

9 $${\boldsymbol{\mu }}={\boldsymbol{\mu }}\_{\bf{m}}{\bf{a}}{\bf{x}}/({\boldsymbol{K}}\_{\boldsymbol{S}})+{\boldsymbol{S}}$$

where; **μ** specific cell growth rate (hr^−1^), **μ**_**max**_ maximum specific cell growth rate (hr^−1^), **S** substrate concentration (g/l), **K**_**S**_ Saturation constant (g/l) = **S** when **μ = **1/2 **μ**_**max**_.

10 $${\bf{X}}{\bf{V}}={{\bf{X}}}_{0}{{\bf{V}}}_{0}\,{{\bf{e}}}^{{\boldsymbol{\mu }}{\bf{t}}},\frac{{\bf{d}}({\bf{S}}{\bf{V}})}{{\bf{d}}{\bf{t}}}=0,\frac{{\bf{d}}({\bf{S}}{\bf{V}})}{{\bf{d}}{\bf{t}}}={\bf{F}}{{\bf{S}}}_{0}-(\frac{{\boldsymbol{\mu }}{\bf{X}}{\bf{V}}}{{{\bf{Y}}}_{{\bf{X}}/{\bf{S}}}}),{\bf{F}}=\frac{{\boldsymbol{\mu }}{{\bf{X}}}_{0}{{\bf{V}}}_{0}{{\bf{e}}}^{{\boldsymbol{\mu }}{\bf{t}}}}{{{\bf{S}}}_{0}{{\bf{Y}}}_{{\bf{X}}/{\bf{S}}}}$$

### Optimization strategy for the biogenic synthesis of nano-MgO

In the present study, Robust Design (Taguchi method) steps applied in a stepwise to optimize the biosynthesis of nano-MgO reaction. To yield a valid final product Taguchi method was set up by using several stages: select the significant variables, designing the accurate matrix, data statistically data analysis and finally validation step by using the optimum levels^23^^–^^25,60^. This study aimed to gain the maximum nano-MgO dry weight which produced by using a different concentration of the intracellular metabolites (reducing/stabilizing agent), Mg (NO_3_)_2_.6H_2_O (precursor) and pH at a different temperature. In this work, L32 Taguchi orthogonal array design (2**4) was chosen for this optimization strategy (4 factors, 2 levels, and 32 runs). After conducting calculation of the average of formed nanoMgO dry weight and signal-to-noise (S/N) ratio (the larger-the-better group) for each reaction condition as designed and F test & ANOVA are also carried out by using the MINITAB 16 software to study the significance of all factors and their relations at specific levels. Finally, a confirmation test was conducted to validate the predicted results produced based on Taguchi’s methods approach with the actual experimental value. In this investigations, the S/N ratios (maximize) are calculated using the Eq. 11. The predicted S/N ratio with the optimal level of the design parameters can be calculated by using Eq. 12.

11 $${\bf{S}}/{\bf{N}}=-10\,\mathrm{log}(\frac{1}{{\bf{n}}})\sum _{{\bf{i}}={\bf{1}}}^{{\bf{n}}}\frac{1}{{{\bf{Y}}}_{{\bf{i}}}^{2}}$$

where **n** is the number of observations and **y** is the observed data; the **S**/**N** ratio is expressed using a decibel scale (**dB**).

12 $$\frac{{\boldsymbol{S}}}{{{\boldsymbol{N}}}_{{\rm{Predicted}}}\,}=\frac{{\boldsymbol{S}}}{{{\rm{N}}}_{{\rm{m}}}}+\,\sum _{{\boldsymbol{i}}=1}^{{\boldsymbol{n}}}|\frac{{\boldsymbol{S}}}{{\boldsymbol{Ni}}}-\frac{{\boldsymbol{S}}}{{{\rm{N}}}_{{\rm{m}}}}|$$

where ***S*/*N***_**m**_ is the total mean ***S*/*N*** ratio and ***S*/*N***_**i**_ is the mean ***S*/*N*** ratio at the optimal level; ***n*** is the number of the parameters.

### Antibacterial efficiency of nano-MgO

Nutrient agar plates were prepared and swabbed with 100 μl of mature broth culture of human pathogenic bacterial strains individually. Firstly, the pathogens sensitivity was tested by using the standard antibiotics^58^ (Amikacin 30 mcg, Ampicilin/sulbactam 40 μg, Ampicillin 10 μg, Cefotaxime 30 µg, Cefoxitin 30 µg, Cephradine 30 μg, Chloramphenicol 30 mcg, Erythromycin 15 mcg, Gentamicin 10 mcg, Nitrofurantion 300 mcg, Norfloxacin 10 mcg, Pefloxacin 5 mcg and Penicillin G 10 U) through disc diffusion method^23–25,43,50,59,60^. Secondly, the minimum inhibitory concentration (a concentration which completely inhibited growth) were calculated by using different concentration of pure nano-MgO solution (5, 10, 15, 20, 25, 30, 35, 40, 45, 50 and 55 µg/ml) through well diffusion method. Then the plates were incubated at 37 °C for 24–48 hr, and the inhibition zones measured in millimeter (mm) of the every well or disc. Analysis of variance (ANOVA) was used to determine whether there was a significant difference between replicates and demonstrate either a significant difference (*p* < 0.05) or no significant difference (*p* > 0.05) for the results obtained. To evaluate the antibacterial activity of 25 µg/ml nano-MgO solution; the comparative study was applied by using 100 mg/ml ampicilin/sulbactam solution as a standard. An inoculum size of test pathogens was added to 50 ml of nutrient broth separately and the bacterial growth adjusted to an OD of McFarland 0.5 (1 × 10^8^ CFU/ml). Subsequently, 50 ml of nutrient broth that supplemented with 25 µg/ml nano-MgO solution and 100 mg/ml ampicilin/sulbactam solution were added to the above flask separately. The negative control bacterial culture was set up without nanoparticles or antibiotic solutions. All the flasks were incubated on rotator shaker (200 rpm) at 37 °C. Eventually, the 1 ml of each sample was collected to determine CFU on nutrient agar plates. Notably, the average number of CFU/ml (log10) was calculated and the final results were analyzed by using Student’s‘t’ test (P-values <5%). Finally, a minimal bactericidal concentration (the lowest nano-MgO concentration that kills 100% of the initial bacterial cells) was calculated by bacterial counting method as described above.

## Results

### Screening of isolated endophytic *Actinomycetes* for biogenic synthesis of active nano-MgO and molecular identification

**Figure 1:**
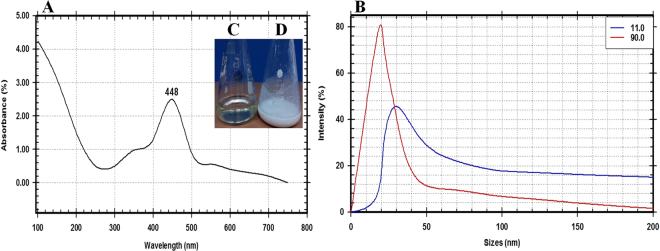
(**A**) UV-visible spectroscopy analysis of the nano-MgO showing SPR peak at 448 nm, (**B**) Nano-MgO particle size curve at two angles 11° and 90°, Digital photograph indicates biogenic synthesis nano-MgO reaction by using endophytic *S*. *coelicolor* strain E72 extract as reducing agent and Mg(NO_3_)_2_.6H_2_O as a precursor: (**C**) Endophytic *S*. *coelicolor* strain E72 extract, (**D**) fabricated nano-MgO.

A total of 10 isolates of endophytic actinomycetes were isolated from leaf tissues of *Ocimum sanctum*. One endophytic isolate out of these ten isolates showed the turbidity change of the cell filtrate after adding the precursor metal salt (1 M Mg(NO_3_)_2_·6H_2_O) as shown in Fig. 1(C,D). This nano-MgO synthesizers isolate was coded as E72. The formation of nano-MgO was confirmed and its properties studied by using important analysis methods (UV-Vis spectroscopy, SEM, TEM, EDX, XRD, PSA, and FTIR) for identifying and characterizing these nanoparticles. A detailed characterization of the prepared nanoparticles was noted after the completed reduction process (24 hr) by an extinction of surface plasmon resonance (SPR), that observed at 448 nm in UV- vis spectrophotometer that confirmed the synthesis of nano-MgO (Fig. 1A). The average particles size found to be 25 nm that measured by using Eq. (1) and confirmed by particle size analyzer at both angles 90° and 11° (Fig. 1B).

**Figure 2:**
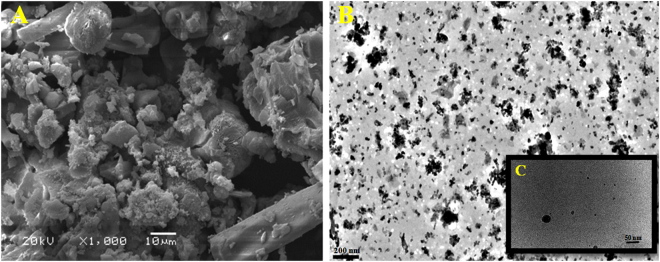
(**A**) SEM analysis of the nano-MgO that bio-fabricated by using endophytic *S*. *coelicolor* strain E72 extract with scale 10 μm. (**B**) and (**C**) TEM images with scales 200 nm and 50 nm respectively.

**Figure 3:**
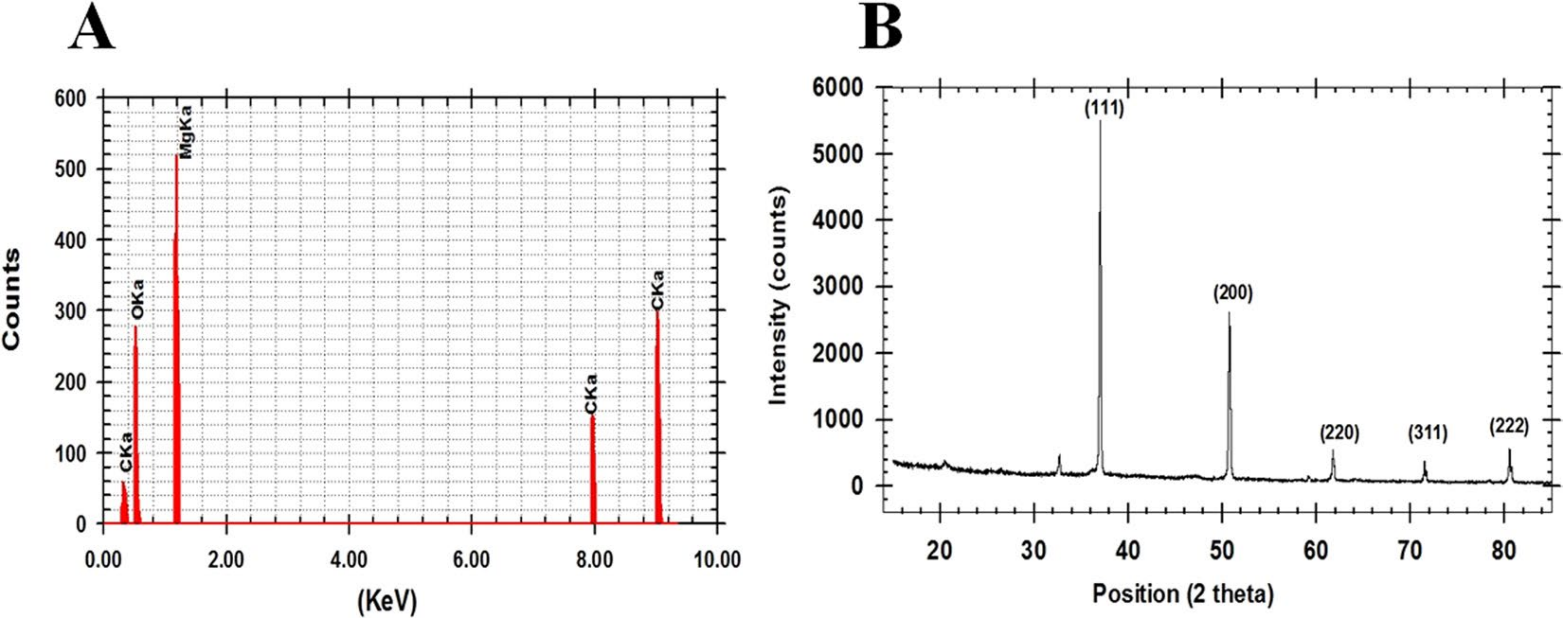
(**A**) Energy dispersive X-ray analysis of the nano-MgO that bio-fabricated by using endophytic *S*. *coelicolor* strain E72 extract and (**B**) XRD spectrum.

The analysis of SEM and TEM data that obtained from micrograph images confirmed the formation of spherical and ellipsoidal nano-MgO shape (Fig. 2). The EDX spectrum that showed a strong metal signal peak of magnesium (Mg) and other peaks of oxygen (O_2_) and a signal of carbon (C) also confirmed the synthesis of nano-MgO (Fig. 3A). The crystalline nature of the nano-MgO was clarified with X-ray diffraction analysis (Fig. 3B). From the XRD pattern, five distinct diffraction peaks were absorbed at 36°, 52°, 62°, 72° and 82° along with miller indices values (111), (200), (220), (311) and (222), respectively and no peak of other phases were found in the X-ray spectrum, arising from an impurity was observed that indicating the nano-MgO obtained through this safe biogenic synthesis method possesses an ultra-pure crystalline phase. This was in agreement with the standard diffraction pattern of a pure cubic phase of nano-MgO which is consistent with bulk MgO crystal (JCPDS Card No. 77–2364, MgO).

**Figure 4:**
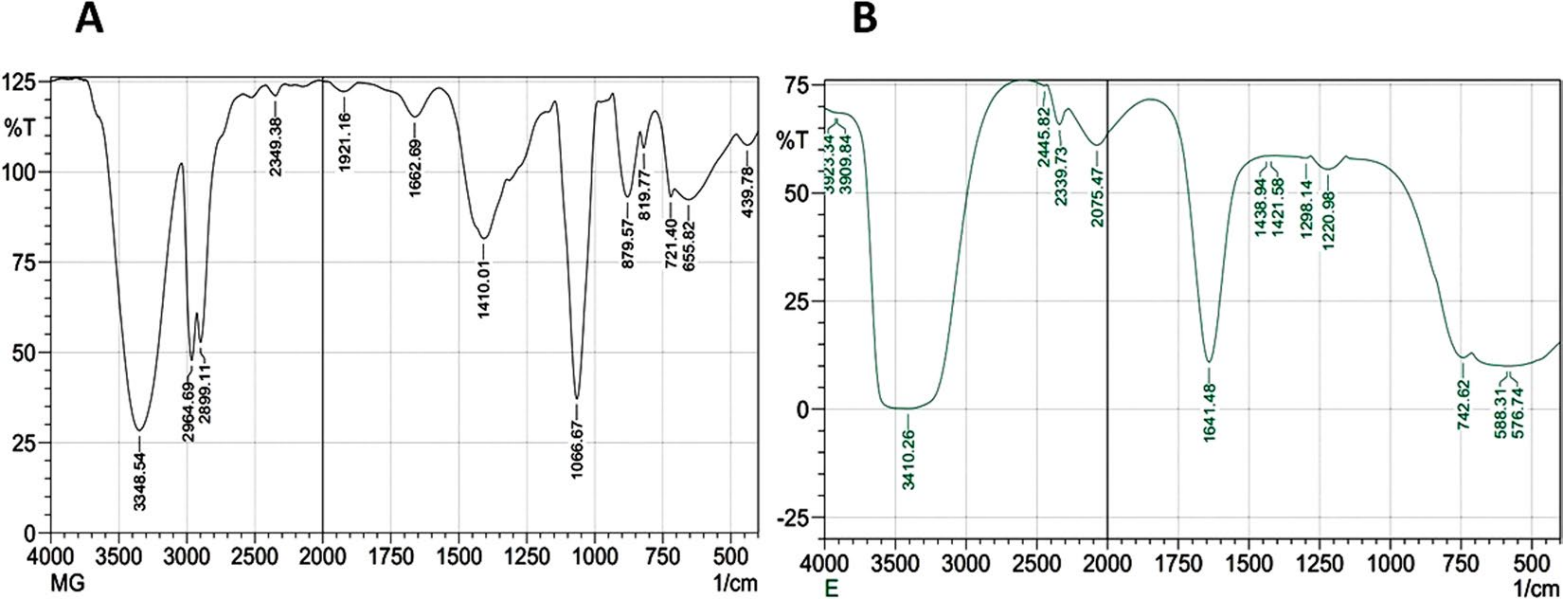
FTIR spectrum of the nano-MgO that bio-fabricated by using endophytic *S*. *coelicolor* strain E72 extract (**A**) and its respective intracellular fraction extract (**B**) in the range of 500–4000 cm^−1^.

To identify the structure of the bonding chemical compound in intracellular fraction before biogenic synthesis of nano-MgO and after this process, the FTIR was measured for the nan-MgO spectrum (capping and stabilizing biomolecules) and the intracellular fraction spectrum (reducing biomolecules) as shown in Fig. 4(A,B). Firstly, the important bands at 439–879 cm^−1^ that observed in the nan-MgO spectrum assigned to the metal–oxygen bonds stretching vibration, which confirm the formation of nano-MgO which agreed well with the UV, EDX and X-ray spectra. Also the intensive peaks were observed in the same spectrum at 1410.84 & 1066.67 cm^−1^ corresponding to the C-N stretching vibration of aliphatic amino acid, 2899 & 2964 cm^−1^ corresponding to C–H stretching of methylene groups of the protein and 3348 cm^−1^ corresponding to N–H stretching of the secondary amide of the protein. In the intracellular spectrum, the intensive peaks of the sample at 3410.26 cm^−1^ corresponding to the C-N stretching vibration of the amide I & amide II bands of the proteins, 1641.48 cm^−1^ corresponding to the stretching vibrations of C=C or the O–H bending mode and 1458.2 cm^−1^ corresponding to the C-N stretching vibration of aromatic and aliphatic amino acid that associated with vibrational mode of H^−^ ion bonded to Mg^2+^ on co-ordination sites. These results confirm that the presence of protein which might have an important role in the biogenic synthesis of nano-MgO.

**Figure 5:**
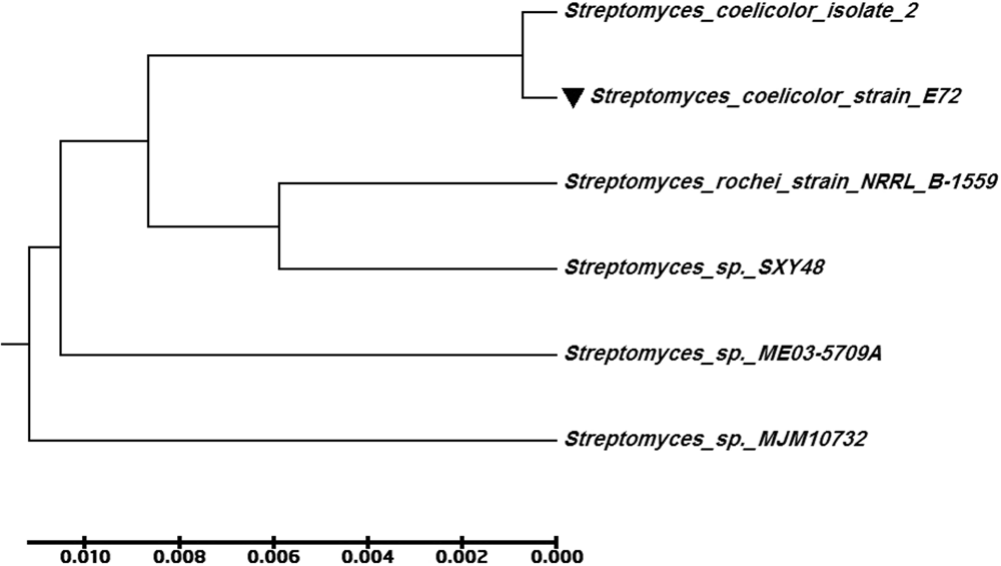
Phylogenetic tree based on 16 S rDNA gene sequence analysis, showing the relationship of endophytic *S*. *coelicolor* strain E72 **(**MF429778) with reference strains (NCBI GenBank) which constructed by using the neighbor-joining method with the aid of MEGA 6.0 program. Sequence divergence is indicated by the scale bar.

In addition to the morphological characterization, the molecular identification was also carried out to confirm the identification of the promising endophytic *Actinomycetes* isolate by using 16 S rRNA gene. The Sanger’s dideoxy nucleotide sequencing of amplified 16 S rRNA gene resulted in 1502 bp nucleotide sequence. The Blastn analyses, pair wise and multiple sequence alignment revealed 98–100% identity with the sequences of *Streptomyces coelicolor* strains and is designated as *Streptomyces coelicolor* strain E72 and has been deposited in NCBI GenBank (Accession Number MF429778). Multiple sequence alignment was carried out using ClustalW2 with default parameters. Phylogenetic tree was constructed by the neighbor-joining (NJ) method with nucleotide pairwise genetic distance as shown in Fig. 5.

The antimicrobial activity of the prepared nano-MgO and some conventional antibiotics were screened and assessed against the tested clinical pathogens as shown in Table (1). The tested human pathogens showed varied sensitivity efficiency against tested antibiotics as well as against prepared nano-MgO. So these results concluded that, nano-MgO showed broad spectrum antibacterial activity against tested multidrug resistant human pathogens.

**Table 1:** Antimicrobial activity of some conventional antibiotics and the prepared MgO nano-powder that fabricated from endophytic Streptomyces sp extract against Gram-positive and Gram- negative human pathogenic bacteria.

Antimicrobial agents	Concentration	Human pathogenic bacteria							
		Gram-negative					Gram-positive		
		*Escherichia Coli*	*Proteus vulgaris*	*Salmonella typhimurium*	*Shigella flexneri*	*Klebsiella pneumonia*	*Staphylococcus aureus*	*Streptococcus pneumonia*	*Bacillus cereus*
Prepared nano-MgO	100 mg/ml	S	R	S	S	S	R	S	S
Amikacin	30 mcg	R	S	R	R	R	S	S	R
Ampicilin/sulbactam	40 mg	R	R	R	R	R	R	R	R
Ampicillin	10 μg	S	R	R	R	R	R	S	R
Cefotaxime	30 µg	S	R	R	R	R	R	S	R
Cefoxitin	30 µg	R	R	S	R	R	R	R	R
Cephradine	30 μg	R	R	S	R	S	R	R	S
Chloramphenicol	30 mcg	R	R	S	R	S	R	R	R
Erythromycin	15 mcg	S	R	R	R	S	R	R	R
Gentamicin	10 mcg	S	S	R	R	R	S	R	R
Nitrofurantion	300 mcg	S	S	R	R	R	S	S	S
Norfloxacin	10 mcg	R	R	S	R	R	S	R	R
Pefloxacin	5 mcg	R	R	R	R	R	R	S	R
Penicillin	10 U	R	R	S	R	S	R	R	R

**R**: Resistant and **S**: Sensitive.

### Screening of the most potent bioactive metabolites for biogenic synthesis of nano-MgO

**Figure 6:**
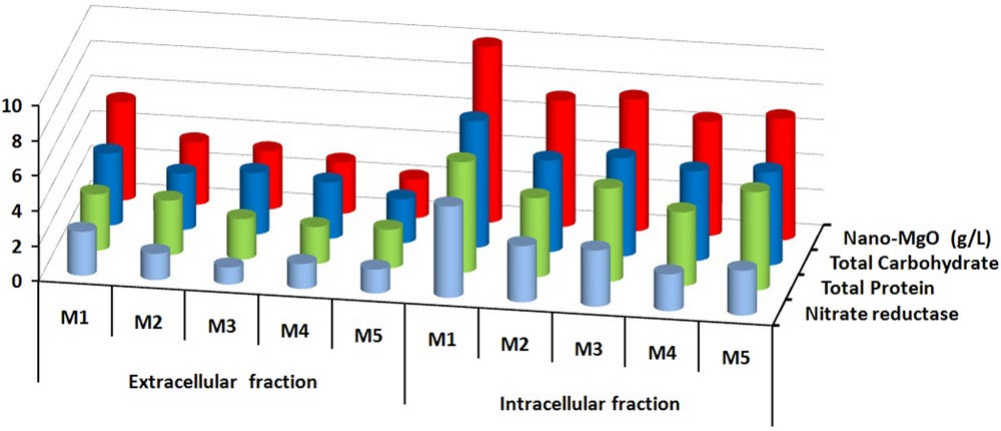
The location of the highest bioactive metabolites (total carbohydrates, total protein and nitrate reductase) and the highest dry weight of nano-MgO that biosynthesized by using the extracellular, and intracellular fractions extracted from endophytic *S*. *coelicolor* strain E72 that cultivated in different five media **(M1**, **M2**, **M3**, **M4 and M5)**.

The modern biotechnological strategies applied in all the following experiments to select the suitable and cheaper cultivation medium that produce the highest *Streptomyces coelicolor* strain E72 mass production and also screening the most potent extracted metabolites that used for biogenic synthesis of nano-MgO. Since, the growth medium considered as one of the most factors that affecting cell density and hence bioactive compounds production during an industrial fermentation process that finally improving nano-MgO biosynthesis. So in the present report, the screening studies including two parameters, the kind of the best media (organic, inorganic and mixed media) and location (extracellular and intracellular fractions) of the active metabolites (nitrate reductase, total protein, and total carbohydrate) that produce the highest nano-MgO dry mass weight. Among the tested media, the Modified Czapek-Dox medium (**M1**) produced significantly higher 2.5 g/l of biomass dry weight and 10 g/l of nano-MgO dry mass weight that recorded by using intracellular fraction. The highest quantitative measurements of these metabolites were detected in intracellular fraction since the highly carbohydrate concentration (7.2 μg/μl), nitrate reductase (5.2 nmol/hr/ml) and protein concentration (6.3 g/l) were recorded as shown in Fig. 6.

### Optimization strategies for the highest *Streptomyces coelicolor* strain E72 biomass production

The microbial cultural requirement (medium components and culturing conditions) for high microbial mass weight and also bioactive compounds production is one of the major cost-effective problem facing industrial producers. So in these investigations statistical-mathematical designs such as Plackett-Burman design (PBD) that followed by Box-Behnken designs (BBD) were used for optimization of the industrial medium components to reduce biomass production costs and hence the prepared nanoparticles production cost by producing the promising bioactive compounds. Firstly, screening process was carried out by using Plackett-Burman design (PBD) to determine which variables significantly affected microbial biomass production based on the 1^st^ order model equation^23–25^. Secondly, response surface methodology was used to study the combined effect among these significant variables and also calculate their optimal levels through Box-Behnken designs (BBD) process based on second-order polynomial equation^23–25,55^.

#### Plackett-Burman design (PBD)

**Figure 7:**
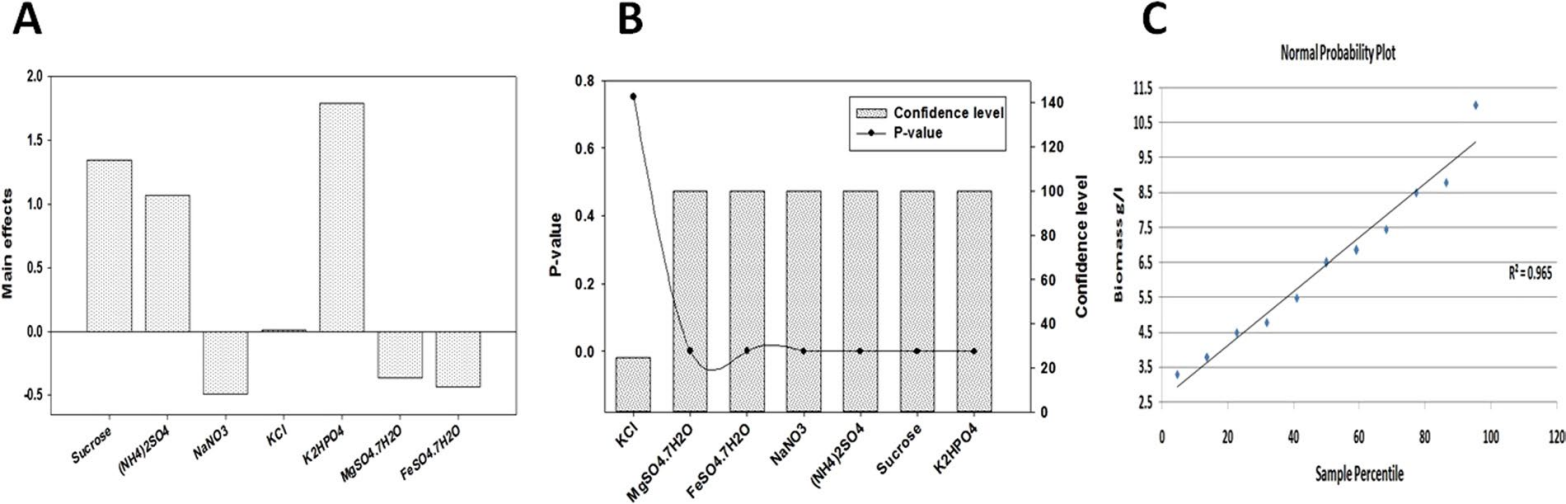
(**A**) Column chart shown the main effect of the variables affecting biomass production of endophytic *S*. *coelicolor* strain E72 according to the results of Plackett-Burman design. (**B**) The Pareto chart represents the *P*-value and confidence level of different variables, (**C**) The normal probability plot of the residuals.

Recently, application of the statistical Plackett-Burman design as a very effective screening tool for optimization of the microbial biomass production reported and discussed in many publications^23–25,55^. In this report, the Plackett-Burman design experiments were conducted in 8 runs to select the significant variables that affected on the final biomass dry weight. Wide variations of biomass production that reflected the importance of the statistical medium optimization step to attain higher biomass production were shown in Table (2). The maximum biomass production (11.05 g/l) was achieved in the run number 2, while the minimum value (3.52 g/l) was observed in the run number 8. The relationship between an independent variables and biomass production is determined by using multiple-regression model and the % confidence level were calculated (Table 3). The main effects of the examined factors on the biomass production were calculated and the normal probability plot of the residuals represented graphically in Fig. 7. The *P-*value from the ANOVA analysis for each variable was determined to analyze the relationship between the variables at 95% or higher confidence level (Fig. 7**)**. The final results revealed that, there is a statistically significant relationship among the tested variables at 99% confidence level. These results indicated that four variables from the seven different independent variable named sucrose, (NH_4_)_2_SO_4_, KCl, and K_2_HPO_4_ affected positively on biomass production, where the three variables named NaNO_3_, MgSO_4_·7H_2_O, and FeSO_4_·7H_2_O affected negatively. The predicted optimum levels of the independent variables were verified and compared with the basal medium setting. The high correlation between the predicted and observed values indicated the validity of the statistical design. The final results indicated that the biomass production can be increased more than 4times when eliminating NaNO_3_ and KCl from the optimized medium. The final composition of optimized medium consisted of (g/l) 40.0 Sucrose, 4.0 (NH_4_)_2_SO_4_, 3.0 K_2_HPO_4_, 0.5 MgSO_4_·7H_2_O, and 0.01 FeSO_4_·7H_2_O. Finally, the significant independent variables named Sucrose (F_1_), (NH_4_)_2_SO_4_ (F_2_) and K_2_HPO_4_ (F_3_) were used for further optimization step by using a Box-Behnken designs (BBD).

**Table 2:** Eight-trial of Plackett–Burman experimental design used for evaluation of seven independent variables along with the endophytic *S*. *coelicolor* strain E72 biomass production.

Trail	Variables (g/l)							Biomass g/l	Predicted Biomass g/l
	Sucrose	(NH_4_)_2_SO_4_	NaNO_3_	KCl	K_2_HPO_4_	MgSO_4_·7H_2_O	FeSO_4_7H_2_O		
1	40	0	0	2	1	1	0.5	4.562	4.623
2	40	4	0	0.5	3	0.5	0.5	11.046	11.053
3	40	4	2	0.5	1	1	0.01	6.564	6.623
4	15	4	2	2	1	0.5	0.5	3.765	3.823
5	40	0	2	2	3	0.5	0.01	8.758	8.823
6	15	4	0	2	3	1	0.01	8.451	8.523
7	15	0	2	0.5	3	1	0.5	4.463	4.523
8	15	0	0	0.5	1	0.5	0.01	3.465	3.523

**Table 3:** Regression statistics, analysis of variance (ANOVA) for Plackett-Berman experimental design results used for optimizing the endophytic *S*. *coelicolor* strain E72 biomass production.

Variables	Coefficients	Standard Error	t Stat	P-value	Confidence level (%)
Sucrose	1.34125	0.025251575	53.115499	1.47E-05*	99.999
(NH_4_)_2_SO_4_	1.06625	0.025251575	42.225089	2.92E-05*	99.997
NaNO_3_	−0.49125	0.025251575	−19.454232	0.000297*	99.970
KCl	0.00875	0.025251575	0.346513	0.751837	24.816
K_2_HPO_4_	1.79125	0.025251575	70.936169	6.17E-06*	99.999
MgSO_4_·7H_2_O	−0.36625	0.025251575	−14.504046	0.000711*	99.929
FeSO_4_7H_2_O	−0.43375	0.025251575	−17.177146	0.00043*	99.957
	**df**	**SS**	**MS**	**F**	**Significance F**
Regression	7	53.6647875	7.6663982	1502.88	2.61E-05
Residual	3	0.015303409	0.0051011		
Total	10	53.68009091			

***Multiple R***, *0*.*99*, ***R Square***, *0*.*99*, ***Adjusted R Square***, *0*.*99*, ***Standard Error***, *0*.*07*, *Significant values, **df**: Degree of freedom, **SS** - sum of squares, **MS**- mean square, **F**: Fishers’s function, **P**: Level of significance.

#### Box-Behnken designs (BBD)

**Figure 8:**
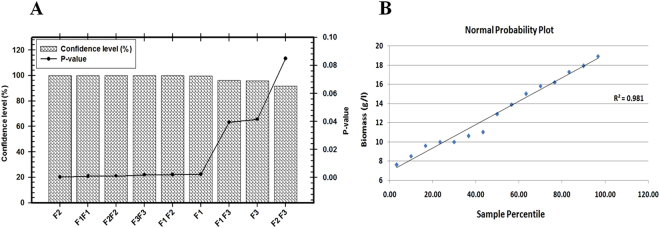
(**A**) Pareto chart represent the *P*-value and confidence level of different variables affecting biomass production of endophytic *S*. *coelicolor* strain E72 according to the results of Box-Behnken design. (**B**) The normal probability plot of the residuals.

In this study, a total of 15 experiments according to a BBD experimental matrix and the final biomass production were performed and summarized in Table (4). The adequacy of the model was checked using ANOVA which was tested using Fisher’s statistical analysis as shown in Table (5). The Model *F*-value of 30.743 with a very low probability value (*P* model > *F* 0.00074) implies the model is significant. The analyzed data revealed that the positive coefficients for some variables and interaction effects increased final biomass production, while other negative coefficients decreased it. By using the degree of significance the analyzed results revealed that the quadratic effect of Sucrose (**F**_**1**_), (NH_4_)_2_SO_4_ (**F**_**2**_) and K_2_HPO_4_ (**F**_**3**_) are significant model terms (*P* value > 0.05) as shown in Fig. 8. Linear coefficients and interaction between three variables are significant (*P* value > 0.05) indicating that there is a significant correlation between three variables that play a major role in increasing the biomass production. In order to evaluate the relationship between dependent and independent variables and to determine the highest biomass production corresponding to the optimum levels of the three tested variables; a second-order polynomial model was proposed to calculate the optimum levels of these variables and defines predicted response (**Y**) in terms of the independent variables:

$$\begin{array}{rcl}{{\bf{Y}}}_{({\rm{biomass}}{\rm{production}})} & = & 17.46+{\bf{1}}.67\ast {{\bf{F}}}_{{\bf{1}}}-{\bf{2}}.67\ast {{\bf{F}}}_{{\bf{2}}}-{\bf{0}}.{\bf{8}}\ast {{\bf{F}}}_{{\bf{3}}}\\  &  & -\,{\bf{3}}.05\ast {{\bf{F}}}_{{\bf{1}}}{{\bf{F}}}_{{\bf{1}}}-{\bf{2}}.90\ast {{\bf{F}}}_{{\bf{2}}}{{\bf{F}}}_{{\bf{2}}}-{\bf{2}}.56\ast {{\bf{F}}}_{{\bf{3}}}{{\bf{F}}}_{{\bf{3}}}\\  &  & -\,{\bf{2}}.50\ast {{\bf{F}}}_{{\bf{1}}}{{\bf{F}}}_{{\bf{2}}}-{\bf{1}}.15\ast {{\bf{F}}}_{{\bf{1}}}{{\bf{F}}}_{{\bf{3}}}+{\bf{0}}.86\ast {{\bf{F}}}_{{\bf{2}}}{{\bf{F}}}_{{\bf{3}}}\end{array}$$

**Table 4:** Box–Behnken experimental design matrix of three variables with actual factor levels corresponding to coded factor levels, actual values, and the mean of *S*. *coelicolor* strain E72 biomass production.

Trial No	Variables (g/l)			Biomass (g/l)	Predicted Biomass (g/l)
	**Sucrose (F**_**1**_ **)**	**(NH**_**4**_ **)**_**2**_ **SO**_**4**_ **(F**_**2**_ **)**	**K**_**2**_ **HPO**_**4**_ **(F**_**3**_ **)**		
**1**	0(50)	0(5)	0(4)	17.3	17.47
**2**	−1(40)	−1(4)	0(4)	10.4	10.00
**3**	0(50)	1(6)	1(5)	9.6	9.39
**4**	0(50)	−1(4)	1(5)	12	13.01
**5**	0(50)	0(5)	0(4)	17.2	17.47
**6**	1(60)	1(6)	0(4)	7.6	8.00
**7**	1(60)	0(5)	1(5)	11.9	11.57
**8**	1(60)	0(5)	−1(3)	15	15.47
**9**	−1(40)	1(6)	0(4)	10	10.54
**10**	−1(40)	0(5)	1(5)	11	10.53
**11**	0(50)	1(6)	−1(3)	10	9.26
**12**	0(50)	−1(4)	−1(3)	16.4	16.34
**13**	−1(40)	0(5)	−1(3)	9.5	9.83
**14**	0(50)	0(5)	0(4)	17.9	17.47
**15**	1(60)	−1(4)	0(4)	18.9	18.36

**Table 5:** Regression statistics, analysis of variance (ANOVA) for Box−Behnken experimental design results used for optimizing the *S*. *coelicolor* strain E72 biomass production.

Variables	Coefficients	Standard Error	t Stat	*P*-value	Confidences level %
F_1_	1.670588235	0.29803	5.60543	0.002498*	99.75
F_2_	−2.670588235	0.29803	−8.96079	0.000289*	99.97
F_3_	−0.8	0.293742	−2.72348	0.041602	95.84
F_1_F_1_	−3.053921569	0.435301	−7.01565	0.000907*	99.91
F_2_F_2_	−2.903921569	0.435301	−6.67106	0.001143*	99.89
F_3_F_3_	−2.562745098	0.435301	−5.88729	0.002009*	99.80
F_1_ F_2_	−2.508823529	0.427457	−5.86918	0.002037*	99.80
F_1_ F_3_	−1.15	0.415414	−2.76832	0.039444*	96.06
F_2_ F_3_	0.864705882	0.403011	2.145616	0.084713	91.53
	**df**	**SS**	**MS**	**F**	**Significance F**
Regression	9	190.9926	21.2214	30.743	0.00074
Residual	5	3.451373	0.690275		
Total	14	194.444			

***Multiple R***, ***0***.***99***, ***R Square***, ***0***.***98***, ***Adjusted R Square*, 0.95**, ***Standard Error***, ***0***.***83***, *Significant values, **df**: Degree of freedom, **SS**- sum of squares, **MS**- mean square. **F**: Fishers’s function, **P**: Level of significance.

The predicted optimal levels of the variables for highest biomass production were calculated and the high-yield medium formulation consisted of (g/l) 48.2 Sucrose, 4.8 (NH_4_)_2_SO_4_, 3.5 K_2_HPO_4_, 0.5 MgSO_4_·7H_2_O, and 0.01 FeSO_4_·7H_2_O. The biomass yield that obtained from the experiment (18.5 g/l) was very close to the predicted one (18.9 g/l) by using the regression model, which proved the validity of the model. The verification revealed a high degree of accuracy of the model of 97.8%, indicating the model validation under the tested medium composition.

### Scaling up fermentation strategies for endophytic *Streptomyces coelicolor* strain E72

In a batch fermentation system(constant operation), all necessary medium components and the microbial inoculum are added at the beginning by using basic controls for pH, temperature, dissolved oxygen, and foam, and the product/s is harvested at the end of this batch run. In fed-batch fermentation system (dynamic operation), the nutrients (carbon source/inducers or whole nutrients) necessary for microbial growth and product formation are added by using one or more feeding system during the operational period and finally the biomass product or the desired product is harvested usually at the end of the operational run^23–25^. Large scale production of the endophytic *Streptomyces coelicolor* strain E72 biomass is a very important step for large scale biosynthesis of nano-MgO system, so in this work scaling up strategy for biomass production and hence nano-MgO production was applied and the growth kinetics studied intensively by using batch and fed-batch operation systems in both of them shake-flask and stirred tank bioreactor (BIOFLO® 310). The behavior of microbial biomass growth can be calculated and described kinetically by using different cultivation mode systems many parameters such as ***X***_***max-Biomass***_, maximal biomass dry weight; ***X***_***max-time***_, time of maximal biomass dry weight; ***dx/dt***, cell growth rate; ***μ***_***max***_, maximal specific growth rate; ***P***_***max-vol***_, maximal volumetric nano-MgO production; ***P***_***max-time***_, time of maximal nano-MgO production; ***Y***_***x/s***_, biomass/glucose consumed; ***Y***_***p/x***_, nano-MgO/biomass; and finally, ***Y***_***p/s***_, nano-MgO/glucose consumed.

#### Batch cultivation in 1 L shake-flask (SF)

**Figure 9:**
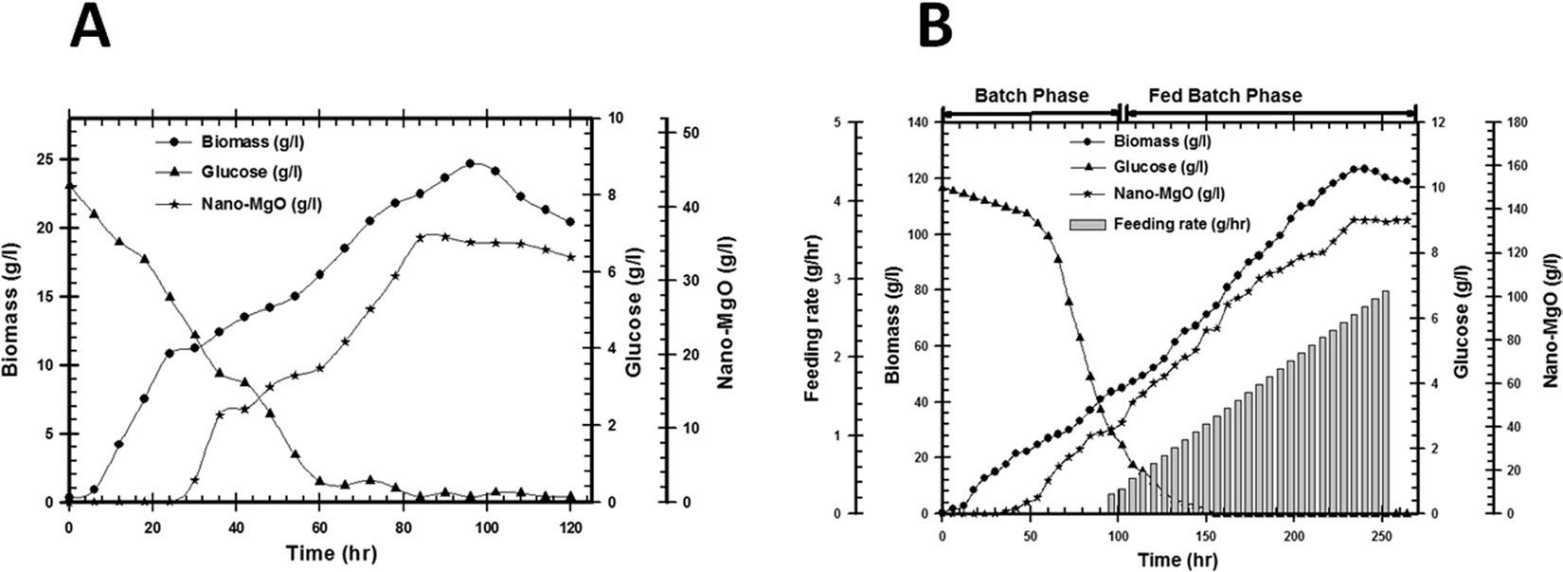
(**A**) Real time monitoring of growth patterns, glucose consumption and biosynthesized nano-MgO for endophytic *S*. *coelicolor* strain E72 that cultivated in 1 L shake flask through batch fermentation mode, (**B**) Real time monitoring of growth patterns, glucose consumption, feeding rate, and biosynthesized nano-MgO for endophytic *S*. *coelicolor* strain E72 that cultivated in 7 L stirred tank bioreactor through exponential pulses fed-batch fermentation mode.

The cultivation in SF was used to detect the time required for the complete consumption of glucose and attainment of the highest biomass & nano-MgO concentration by studying the growth kinetics. The endophytic *Streptomyces coelicolor* strain E72 pre-culture was inculcated in 1 L shake flask (working volume 500 ml). As shown in Fig. 9A, an initial glucose concentration was used gradually and consumed after 84 hr (0.14 g/l). By using kinetics measurements for produced microbial cells, the highest biomass (***X***_***max-Biomass***_) and nano-MgO (***P***_***max-vol***_) production were 24.65 and 35.95 g/l respectively after 118 hr as shown in Table (6).

**Table 6:** Growth conditions and Kinetic parameters for biomass producton, nano-MgO production, and glucose consumption during the endophytic *S*. *coelicolor* strain E72 cultivation in different cultivation scales and mode of operation in shake flask and in stirred tank bioreactor.

Parameters	Shake flask	Batch fermentation	Fed- Batch fermentation
**Growth conditions**			
Container volume (ml)	1000	7000	7000
Working Volume (ml)	500	2500	5500
pH	Un-controlled	6.9	
Aeration (v/v)	Un-controlled	Adjusted to let the dissolved O_2_ level did not below 40%	
Agitation (RPM)	200	Adjusted to let the dissolved O_2_ level did not below 40%	
Feeding rate (g/l/hr)			0.42
Fermentation period (hr)	118	96	192
**Yield coefficients**			
Y_x/s_ (g/g)	2.465	4.36	12.33
Y_p/x_ (g/g)	0.46	0.89	1.10
Y_p/s_ (g/g)	3.595	3.9	13.53
**Growth and glucose consumption parameters**			
X_max-Biomass_ (g/l)	24.65	43.6	123.3
X_max-time_ (h)	96	96	240
dx/dt (g/l/h)	0.058	0.056	0.057
μ_max_ (1/h)	0.052	0.32	0.024
**Nano-MgO production parameters**			
P_max-vol_ (g/l)	35.95	39	135.3
P_max-time_ (h)	90	90	246

#### Batch cultivation in 7 L stirred tank bioreactor (BF)

In this experiment, biomass and nano-MgO production kinetics were calculated and studied during batch cultivation in 7 L stirred tank bioreactor using the same medium composition and inoculum concentration as used in the shake flask experiment. The pre-culture inculcated in 7 L stirred tank bioreactor (working volume 2500 ml). During the batch cultivation, glucose was completely consumed after 96 hr. In this step, the growth kinetics measurements indicated that; ***µ***_***max***_ was 0.03, ***X***_***max***_ was 43.6 g/l, P_**max**_ was 39 g/l; and incubation period was 96 hr as shown in Fig. 9B and Table 6. These results indicated that the batch fermentation data was higher than the shake flask experimental data at different stages of cell growth due to the controlled conditions in stirred tank bioreactor (agitation, airflow and pH).

#### Fed-batch cultivation using exponential pulses feeding strategy with sucrose feeding in 7 L stirred tank bioreactor (FBF)

In this step, cultivation was run under the same conditions of batch culture for the first 96 hr. After that time, feeding was started at an exponential rate of 0.84 g/hr using a concentrated sucrose solution (700 g/l) as shown in Fig. 9B. The pattern of glucose consumption rate and biomass production indicated that when glucose became limited in the culture, biomass dry weight decreased immediately as function of cell death, so the feeding strategy started before this microbial growth stage. The results showed that the fed-batch culture using pulses sucrose feeding system that added exponentially produced biomass concentration (***X***_***max-Biomass***_) 123.3 g/l and nano-MgO (***P***_***max-vol***_) 135.3 g/l after 192 hr (Table 6**)**. The calculated yield coefficients ***Y***_***p/x***_ and ***Y***_***p/s***_ indicated that the grown cells in this culture showed the highest physiological activities depending on the highest substrate conversion for bioactive metabolites production when compared with SF and BF systems.

### Optimization strategy for the nano-MgO biogenic synthesis reaction

**Figure 10:**
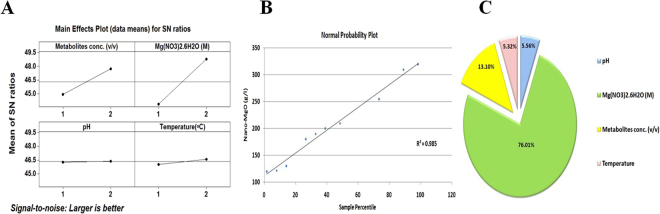
(**A**) Response graph of S/N ratio for larger-the-better analysis of nano-MgO production according to the Taguchi*’s* experimental results. The horizontal axis shows the different levels of each significant factor. The lines represent the trend of each factor with respect to different levels. (**B**) The normal probability plot of the residuals. (**C**) Percent distribution of each factor to the response for nano-MgO production.

Genichi Taguchi has developed an attractive effective tool for optimization and development the high quality industrial production process at a low cost^23–25^. In this design the factor levels (the inner array) are identified in accordance with numbers 1, 2…., and so on that tested against various combinations of the noise factors in the outer array so this design called design of an orthogonal array (signal-to-noise ratio” S/N”). The **S/N** ratio is expressed using a decibel scale (**dB**)^25^. In this work, the biosynthesis of nano-MgO via a simple, fast and eco- friendly method using the Taguchi design (**TD**) was evaluated. Taguchi’s model has several arrays to be used that can be chosen based on the experiment’s requirement. Design of an orthogonal array (OA) coded as ***L***_***n***_ ***(m***^***k***^**)** where ***n***: number of lines, ***m*:** number of parameter levels, and ***k*:** number of parameters. In this work, thirty two experiments were conducted at different setup of four parameters coded based on **L**_**32**_ (**2**^**4**^) OA design to calculate the production of nano-MgO and their corresponding S/N ratio values that listed in Table 7. An ANOVA, the normal probability plot of the residuals and an *F*-test are used to analyze the experimental data. Moreover, in multiple linear regression analysis, there is a value of R^2^ which is the regression coefficient where R^2^ is 0.99 for the models, which indicate that the fit of the experimental data is satisfactory as shown in Table (8). Based on the ANOVA analysis of the S/N ratio value and calculation of main effect for nano-MgO biosynthesis parameters by using factor level, the highest delta is the top rank as shown in Fig. 10. So the ranked variables with actual values from the most to the less impactful parameters that affect nano-MgO biosynthesis performance was obtained with Metabolites conc. (100%), Mg (NO_3_)_2_.6H_2_O (1 M), Temperature (100 °C) and pH (7.0). Finally, a confirmation test for this design is necessary in order to verify the optimum conditions of nano-MgO biogenic synthesis design. The optimal conditions are set for all factors and a selected number of experiments are run under this defined condition. The result from the confirmation experiment (Mean: 320 ± 8.07 [g/l] and S/N Ratio: 50.92 ± 4.90 [dB]) is compared with the predicted one (Mean: 315 ± 7.07 [g/l] and S/N Ratio: 49.96 ± 1.95 [dB]) based on the variables and their levels tested so there is a very good agreement between the predicted and experimental value is observed. From these results, the nano-MgO biosynthesis can be increased (2.4 times larger than basal condition) and improved through the Taguchi method approach.

**Table 7:** Taguchi’s L_32_ (2^4^) Orthogonal array design experimental setup for final optimization of nano-MgO biogenic synthesis reaction by using the endophytic *S*. *coelicolor* strain E72 intracellular extract.

Exp	Coded levels of independent variables				Nano-MgO (g/l)		S/N ratio [dB]
	Metabolites conc	Mg(NO_3_)_2_.6H_2_O	pH	Temperature	Actual value ± SD	Predicted value	
1	1	1	1	1	120 ± 00.00	120.72	41.58
2	1	1	1	1	120 ± 00.00	120.72	41.58
3	1	1	1	2	130 ± 00.00	133.03	42.28
4	1	1	1	2	130 ± 00.01	133.03	42.28
5	1	1	2	1	122 ± 00.00	123.53	41.73
6	1	1	2	1	122 ± 00.01	123.53	41.73
7	1	1	2	2	133 ± 00.00	135.84	42.48
8	1	1	2	2	133 ± 00.01	135.84	42.48
9	1	2	1	1	235 ± 21.21	237.28	47.37
10	1	2	1	1	235 ± 21.22	237.28	47.37
11	1	2	1	2	250 ± 00.00	249.59	47.96
12	1	2	1	2	250 ± 00.01	249.59	47.96
13	1	2	2	1	250 ± 00.00	240.09	47.96
14	1	2	2	1	250 ± 00.01	240.09	47.96
15	1	2	2	2	252.5 ± 03.53	252.41	48.04
16	1	2	2	2	252.5 ± 03.54	252.41	48.04
17	2	1	1	1	185 ± 07.07	184.16	45.33
18	2	1	1	1	185 ± 07.08	184.16	45.33
19	2	1	1	2	205 ± 07.07	196.47	46.23
20	2	1	1	2	205 ± 07.08	196.47	46.23
21	2	1	2	1	185 ± 07.07	186.97	45.33
22	2	1	2	1	185 ± 07.08	186.97	45.33
23	2	1	2	2	200 ± 00.00	199.28	46.02
24	2	1	2	2	200 ± 00.01	199.28	46.02
25	2	2	1	1	300 ± 00.00	300.72	49.54
26	2	2	1	1	300 ± 00.01	300.72	49.54
27	2	2	1	2	310 ± 00.00	313.03	49.83
28	2	2	1	2	310 ± 00.01	313.03	49.83
29	2	2	2	1	300 ± 00.00	303.53	49.54
30	2	2	2	1	300 ± 00.01	303.53	49.54
31	2	2	2	2	315 ± 07.07	315.84	49.96
32	2	2	2	2	315 ± 07.08	315.84	49.96
**Level**	**(v/v)**	**(M)**		**(**°C**)**			
1	10%	0.5	5	50			
2	100%	1	7	100

**Table 8:** Statistical analysis of Taguchi design showing coefficient, *t*-test values, *P*-values and confidence level (%) for variables affecting on nano-MgO biogenic synthesis reaction by using the endophytic *S*. *coelicolor* strain E72 intracellular extract.

Variables	Coefficients	Standard Error	T Stat	*P*-value	
Intercept	−74.4063	6.898715	−10.7855	2.74E-11	
Metabolites conc. (v/v)	63.4375	2.268283	27.96718	1.79E-21*	
Mg(NO3)2.6H2O (M)	116.5625	2.268283	51.38798	1.8E-28*	
pH	2.8125	2.268283	1.239924	0.225672	
Temperature(°C)	12.3125	2.268283	5.428113	9.66E-06*	
	**df**	**SS**	**MS**	**F**	**Significance F**
Regression	4	142165.1	35541.28	863.4723	4.66459E-28
Residual	27	1111.344	41.16088		
Total	31	143276.5			

***Multiple R***, *0*.*99*, ***R Square***, *0*.*99*, ***Adjusted R Square***, *0*.*99*, ***Standard Error***, *6*.*41*, *Significant values, **df**: Degree of freedom, **SS**- sum of squares, **MS**- mean square, **F**: Fishers’s function, **P**: Level of significance.

**Figure 11:**
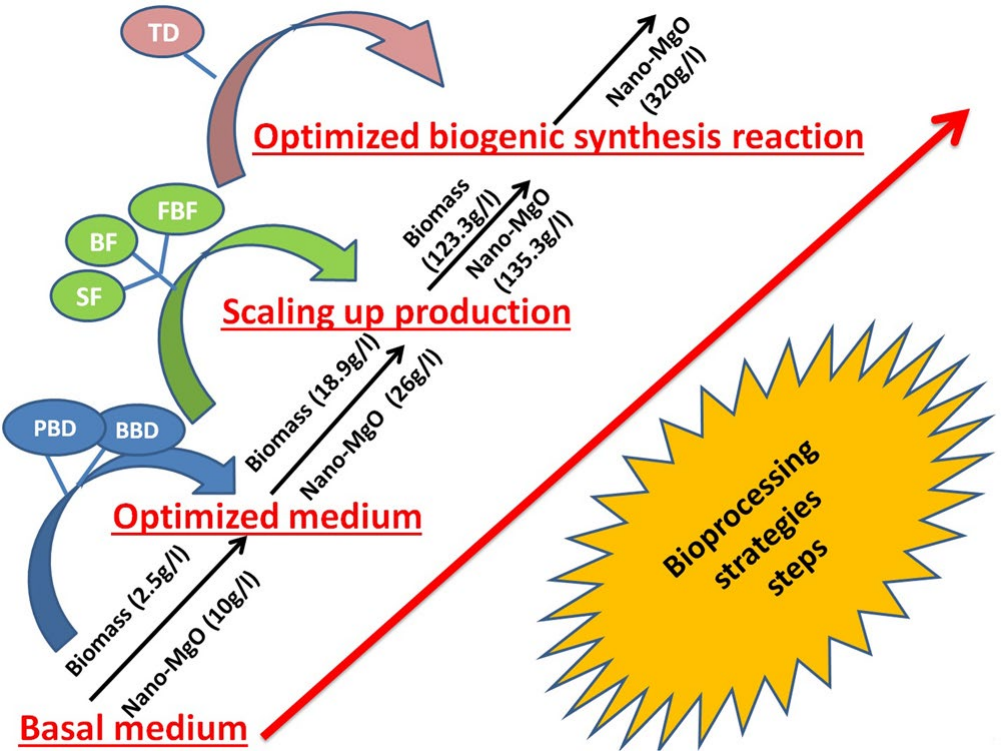
Summary of the maximum biomass weight of endophytic *S*. *coelicolor* strain E72 and the highest nano-MgO production during the used bio-processing strategies after applying Plackett-Burman (PB), Box-Behnken designs (BB) and Taguchi*’s* experimental design (TD) and large scale batch cultivation (BF) in 1 L shake flask (SF), and exponential pulses fed-batch fermentation mode in 7 L stirred tank bioreactor (FBF).

Finally, this report considered as the first report that demonstrated for using the potential of nanobiotechnological strategies to enhance the biogenic synthesis of nano-MgO by using active metabolites extracted from endophytic *Streptomyces coelicolor* via semi-industrial scale bioreactor. The experimental designs strategies and statistical inference results indicated that both biomass and nano-MgO productions were higher by using the optimized culture and the optimized biosynthesis reaction respectively compared to the corresponding uncontrolled conditions by more than 49 and 30 times, respectively through PBD, BBD, pulses feeding fermentation strategy and TD as summarized in the Fig. 11.

### Evaluation of antibacterial activity *in vitro* for biogenic synthesized nano-MgO

**Figure 12:**
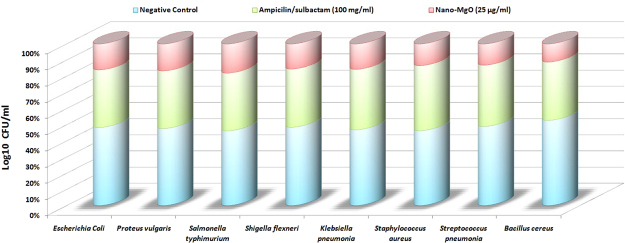
The comparative study among the 25 µg/ml nano-MgO solution (average of MICs) and 100 mg/ml ampicilin/sulbactam solution as a standard antibiotic with negative control (native microbial culturing) by calculating the antibacterial activity through Log10 CFU/ml.

Antibacterial activity test was performed by using different concentrations of prepared nano-MgO against some human pathogenic bacteria to determine the large inhibition zone was calculated and the lowest concentration of nano-MgO that inhibits or kills 100% of the initial bacterial cells were also checked as shown in Table (9). The nano-MgO concentrations that formed large inhibition zones were varied from 5 to 55 μg/ml which showed the highest antibacterial activity against *Shigella flexneri* (108 mm at 25 μg/ml) followed by *Staphylococcus aureus* (102 mm at 15 μg/ml), *Streptococcus pneumonia* (89 mm at 40 μg/ml) and *Klebsiella pneumonia* (85 mm at 20 μg/ml). The lowest concentration of nano-MgO that inhibits of the initial bacterial cells was also varied in descending order, 15, 20, 25 and 40 μg/ml. The bactericidal efficiency also varied from 35 to 55 μg/ml. The comparative study performed to evaluate the final antibacterial activity of 25 μg/ml nano-MgO solution (average of MICs) and ampicilin/sulbactam solution (100 mg/ml) as a standard antibiotic with negative control (native microbial culturing) as shown in Fig. 12. By calculating the Log10 CFU/ml, the results indicated that the nano-MgO solution (25 μg/ml) inhibits 90% of the pathogenic living cells compared with ampicilin/sulbactam solution (100 mg/ml) that killed 40% of the same pathogenic living cells. Finally, these results revealed that nano-MgO showed a strong inhibition activity against anti-multidrug-resistant pathogens not only gram negative but also against gram positive.

**Table 9:** Detection of the maximum inhibition zone produced from bio-application of biogenic synthesized nano-MgO as antibacterial agent against some human pathogenic bacteria *in vitro*.

Human pathogenic bacteria	Nano-MgO concentration (µg/ml)												
	Zone of inhibition (mm)											MIC	MBC
	5	10	15	20	25	30	35	40	45	50	55		
*Escherichia coli*	2 ± 0.052	15 ± 5.5	36 ± 12.3	42 ± 15.3	56 ± 1.73	36 ± 8.6	38 ± 3.6	20 ± 3.6	14 ± 1.3	10 ± 3.4	4 ± 0.3	25	50
*Proteus vulgaris*	3 ± 0.52	22 ± 2.3	45 ± 2.36	75 ± 2.5	69 ± 2.14	68 ± 3.6	59 ± 3.6	42 ± 6.3	25 ± 10.3	12 ± 2.5	8 ± 4.3	20	55
*Salmonella typhimurium*	2 ± 0.10	18 ± 1.36	36 ± 5.69	53 ± 0.3	62 ± 1.38	59 ± 2.6	52 ± 4.6	41 ± 3.6	39 ± 1.5	35 ± 15.3	30 ± 4.9	25	50
*Shigella flexneri	4 ± 1.3	10 ± 2.3	15 ± 1.3	25 ± 5.3	108 ± 10.53*	100 ± 12.3	95 ± 1.3	92 ± 10.3	89 ± 3.6	82 ± 0.05	75 ± 15.3	25	40
*Klebsiella pneumonia*	1 ± 0.03	36 ± 4.5	72 ± 3.69	85 ± 6.2	63 ± 3.21	58 ± 2.3	45 ± 3.5	30 ± 12.4	21 ± 12.3	10 ± 2.4	7 ± 2.1	20	45
*Staphylococcus aureus*	2 ± 1.3	52 ± 10.5	102 ± 10.3	95 ± 4.5	81 ± 9.14	75 ± 1.3	74 ± 2.3	69 ± 9.6	62 ± 2.3	25 ± 10.8	15 ± 5.2	15	35
*Streptococcus pneumonia*	3 ± 0.2	12 ± 0.05	25 ± 8.3	35 ± 2.21	40 ± 2.32	55 ± 4.3	65 ± 4.6	89 ± 7.4	78 ± 2.5	71 ± 12.6	62 ± 3.2	40	50
*Bacillus cereus*	3 ± 0.5	15 ± 0.03	25 ± 0.05	45 ± 3.6	75 ± 6.3	62 ± 6.3	45 ± 0.8	21 ± 5.2	15 ± 4.6	9 ± 3.9	7 ± 1.3	25	45

*The maximum inhibition zone.

## Discussion

The traditional methods of synthesis of nano-metal oxide are chemical and physical techniques that are usually expensive and potentially hazardous to the environment and human health. During the past decade, many biological systems (bacteria, yeast, and fungi) has been demonstrated and developed for biosynthesis of nano-metal oxide by using extracted proteins and metabolites^23–25,61^. For industrial production of nano-metal oxide; the safety, the low cost of microbial cultivation, short biosynthesis reaction time, and the ability to scale-up production; make *Streptomyces sp*., an attractive factory for a biogenic synthesis of nanoparticles that used for therapeutic applications^17,50,60,62^. Recently, biogenic synthesis of nanoparticles has taken an important stage in the modern industrial pharmaceutical sector, so exploring novel eco-friendly bioactive metabolites extracted from safe potential bio-factories that possess low toxicity was studied in this work.

There are organic (bacteriocins, enzymes, etc) and inorganic (metal and metal oxide, etc) compounds were reported as proficient antibacterial agents^10,40^. But inorganic antibacterial agents have attracted for bacterial control due to their stability under harsh industrial processing conditions^8^. Metal oxide nanoparticles have considerable potential as a new class of antimicrobial agents that have been increasingly studied for their potential industrial applications in food safety and health care. Inorganic metal oxides nanoparticles that have high stability and discipline sizes, shapes, surface properties, & chemical compositions were used recently as antimicrobial agents. And due to their large surface area that interact with pathogens cells; these nanoparticles have a broad spectrum antimicrobial activity. Since some studies suggested that higher antimicrobial activities depends upon not only the nanoparticles sizes, shapes, chemical composition, and surface properties but also varies with pathogens species. Nano-MgO (nano-metal oxide) is very interesting due to its strong antimicrobial activities, furthermore its structure, surface properties, high thermal stability and the simplicity of its preparation.

It is known that the medicinal plants have a novel natural compounds and endophytes population (microbial cells has been found to be associated with the internal tissues of a plant without causing detectable disease) which used for industrial biotechnological applications^13,28,38^. Endophytes possess the potential to produce eco-friendly novel primary and/or secondary metabolites, which can be attracted an attention in pharmaceutical industries. The biosynthesis of silver nanoparticles as an antimicrobial agent by using the epiphytic *Streptomyces coelicolor* klmp33 pigment was reported by Manikprabhu^15,16^. By gathering sufficient knowledge, there are no studies on isolation of the endophytic *Streptomyces* from the medicinal plants and used it for nano-MgO biosynthesis. So, reliable biogenic synthesis of nano-MgO by using safe metabolites that extracted from the less explored endophytic *Streptomyces sp*. which isolated from the healthy medicinal plant (*Ocimum sanctum)* was characterized, evaluated, and developed through this study for environmental, and health safety therapeutic applications (antimicrobial agent). In the present investigations, different bioprocessing strategies used to maximize the *Streptomyces* biomass and nano-MgO yield in short reaction time. Through this work; endophytic *Streptomyces coelicolor* strain E72 selected as a proficient factory for the biogenic synthesis of nano-MgO that used as a promising antibacterial agent.

There are few reports on the synthesis of MgO nanoparticles as antimicrobial agent alone, mixed with other metal oxide nanoparticles or mixed with antibiotics against pathogenic bacteria and fungi^5,9^. In nano-medicine, the biogenic synthesis method that used to fabricate the inorganic metal oxide nanoparticles is a new safe method compared to the electrochemical fabrication methods^6^. The physicochemical properties of nanoparticles (particle size, surface area, and chemical composition/structure) are important factors that contribute significantly to the toxicity of these nanoparticles. Sulfur-containing proteins, carbohydrates, saponins, phenolic compounds, quinones free amine groups and NADH-dependent nitrate reductase are reported as bio-reduction and/or bio-stabilizing agent (microbial cell-free extracts) that found to be responsible for the biogenic synthesis of metal oxide nanoparticles from metal salts^3,43,63^. In this report, the intracellular fraction that extracted from endophytic *Streptomyces coelicolor* strain E72 used as nano-MgO synthesizers. This fraction that contains 7.2 μg/μl of carbohydrate, 6.3 g/l of protein and 5.2 nmol/hr/ml of nitrate reductase used to produce multi-surface shaped nano-MgO (spherical and ellipsoidal) with diameter ~25 nm that have surface plasmon resonance at 448 nm. All of these results are strongly supported by former reports^5,6,40,43^.

A killing of multidrug-resistant human pathogens considered as a crucial challenge for public health and required a new noble biotechnological strategy. Nano-antimicrobial agents are useful tools used for a wide variety of multidrug-resistant human pathogens. Several mechanisms have been reported previously for the antimicrobial therapeutic agent’s activity^4^. Nanoparticle toxicity may be caused on the cellular surface (interacts with membrane lipids by electrostatic interaction) or/and inside the cellular organelles (the production of toxic oxygen radicals) which loss of cell membranes integrity, damage DNA, or cell proteins, and finally inhibits the bacterial growth^13,22^. Others suggested that nanoparticles toxicity caused by increasing intracellular reactive oxygen species (ROS) levels and/or the levels of inflammatory mediators followed by molecular and cellular events that cause oxidative stress, severe genotoxicity (frame shift mutation) and then cell death^7^. The mechanism for ROS generation (irreversible damage) is different for each nanoparticle and the exact mechanism for ROS generation is incompletely understood^14^. As a general, the exact mechanisms of toxicity that caused by nanoparticles treatment are very complicated and are not understood completely^12,64^.

The endophytic *Streptomyces sp*. has a proficient capacity to produce a large variety of different low toxicity bioactive metabolites that have a wide spectrum of important industrial activities and continue to be screened for new low-cost commercial bioactive substances. Metabolic activities inside the cell are extremely sensitive to the ambient environment (nutritional media and culturing conditions) and regulated at various levels, so it is very important to study and understand the nutritional and environmental factors affecting cell metabolism^45^. The microbial nutrition environment plays a significant role in the biomass production and hence in different metabolites production via the fermentation process. Evaluation, selection, and optimization of the culturing media are essential steps that prior the semi-pilot or pilot production plans for biomass production and hence for bioactive metabolite production^65^. Advanced statistical medium optimization technique has been proved as the effective optimization strategies that might be useful for the overproduction of many bioactive metabolites in the large scale fermentation process^1^. The successful industrial bioprocessing strategies depend on the product cost that could be reduced by using cheaper carbon/nitrogen sources (growth inducer) and/or by increasing the bioactive metabolites productivity and reduce the loss of unused substrate^66^. So the advanced statistical medium optimization strategies help in reducing the desired bioactive metabolites cost^2^.

The extremely low biomass yields, the activity of the metabolites and long incubation period have restricted its further large-scale production and final marketing strategies^15,16,30,67^. That is mean; there is a relationship between the medium compositions & process parameters and maximum bioactive metabolites & biomass production. The microbial growth and accumulation of bioactive metabolites are strongly influenced by carbon sources, nitrogen sources, various inorganic salts and trace elements. So, to achieve the high-value of the bioactive metabolic compounds, it is necessary to evaluate an efficient industrial fermentation medium^20,34,42,55^. Important attention is paid to the selection of carbon source and nitrogen source because of their role in the microbial biomass yield and hence the formation of bioactive metabolites. There are some carbon/nitrogen sources have the better influence on the biomass growth but others reduced production of many bioactive metabolites^21,68–70^. For potential industrial large-scale fermentation, the choice of the carbon/nitrogen sources also depends on their availability on the market and prices. An excellent carbon source (the monosaccharide, disaccharide, or polysaccharide) and nitrogen sources (organic, inorganic or mix) have been added as an energy source to influence the microbial growth and the formation of several metabolic compounds. For *Streptomyces* species there is diversity in carbon/nitrogen sources can be used^10,27,41,66,71,72^. So in the present report, the primary screening studies including two influenced parameters selection the suitable and cheaper cultivation medium for *Streptomyces coelicolor* strain E72 mass production and the most potent extracted metabolites that used for the biogenic synthesis of nano-MgO. The final results showed that the *Streptomyces coelicolor* strain E72 was able to grow in all the tested carbon/nitrogen sources but modified Czapek-dox medium (M1) was selected as the original fermentation medium among the tested media (that contained different carbon/nitrogen sources). Since sucrose and (NH_4_)_2_SO_4_/NaNO_3_ were the carbon/nitrogen sources that supported the maximum growth rate as well as the highest bioactive metabolites production at the same time. These results indicated that the highest bioactive metabolites that used for the biogenic synthesis of nano-MgO depend on the *Streptomyces coelicolor* strain E72 growth medium constituents. Different statistical experimental designs used in this stage to optimize the basic fermentation medium that selected via this work. Combination of the first-order model PBD (screening of the variables) and the second-order model BBD (estimate the optimal value and the interactions effects among a set of controlled experimental factors) were applied for optimization of different medium components for biomass and bioactive metabolites (that used for biogenic synthesis of nano-MgO) production by *Streptomyces coelicolor* strain E72. The final results indicated that the biomass production can be increased more than 7times when eliminating NaNO_3_ and KCl from the optimized medium and the high-yield new medium formulation consisted of (g/l) 48.2 Sucrose, 4.8 (NH_4_)_2_SO_4_, 3.5 K_2_HPO_4_, 0.5 MgSO_4_·7H_2_O, and 0.01 FeSO_4_·7H_2_O. In this work, the inorganic nitrogen source ((NH_4_)_2_SO_4_) and disaccharide carbon source (sucrose) are preferred for the highest endophytic *Streptomyces coelicolor* strain E72 biomass and bioactive metabolites production compared to organic nitrogen and polysaccharide carbon sources this conclusion opposite to the earlier reports^41,45,65,73^. There are studies recorded that the specific metabolites production doubled when nitrate was replaced with ammonium, and the biomass yield increased many times^46,48,68,70^. Most of the growth media that reported earlier contained a mix of complex organic and/or inorganic nitrogen sources, viz the proficient *Streptomyces* medium cannot be generalized^1,47,67,74^. Finally, the endophytic *Streptomyces coelicolor* strain E72 cultivated by using cost-effective industrial fermentation medium (inexpensive substrates). Until now, *Streptomyces coelicolor* is a proficient applied strain of the genus *Streptomyces* that extracts different bioactive metabolite compounds in clinical field^71^. The promising strain was characterized by a high-yield biomass production by using inexpensive nutrients^44^. Wentzel^71^ reported that the presence of D-glucose and/or L-glutamate was preferred as carbon sources used in *Streptomyces coelicolor* cultivation strategy. Dzhavakhiya^44^ concluded that the highest-biomass yield of *Streptomyces virginiae* and hence the virginiamycin yield was recorded by using sucrose as a carbon source instead of more expensive glucose or D-maltose but complex carbohydrates (starch) shown a lower effect.

Realize biomass productivity via scaling-up strategies strongly depends on ideal fermentation conditions (dissolved oxygen, gas flow, agitation, temperature, pH, etc.) and fermentation modes (batch, substrate fed-batch, whole nutrient fed-batch, etc.)^51,75,76^. Carbon catabolite repression the biosynthesis of many bioactive metabolites is a big problem caused by the carbon source (sucrose) during the *Streptomyces virginiae* cultivation. To solve this problem, a fed-batch fermentation mode was used by maintaining the substrate concentration at a very low level (below a critical level) to improve the highest biomass and metabolites yields^44,71^. There are several publications and patents using *Streptomyces* strain as antibiotics bio-factory that industrial cultivated by using different carbon/nitrogen sources and minor elements (the medium components) via different optimized fermentations modes (optimization of fermentation conditions and/or addition of precursors or regulators)^46,48,51,68,70,75,76^. To the best of our knowledge, this is the first report to evaluate endophytic *Streptomyces coelicolor* biomass production (123.3 g/l) to extract the highest bioactive metabolites that used for the biogenic synthesis of nano-MgO (135.3 g/l) using exponential sucrose pulses feeding strategy after 192 hr in semi industrial scale bioreactor (7 L).

The reduction process of metal ions to produce metal/metal oxide nanoparticles is affected by various factors such as exogenous biometrics, the reaction mixture pH, incubation temperature, reaction time, and electrochemical potential of a metal ion^39,62^. So, the factors that affect the morphology, size, and yield of metal nanoparticles studied and optimized for the proficient fabrication of metal oxide nanoparticles. Terpenoids, polyphenols, sugars, alkaloids, phenolic acids, peptides, an aldehyde group, proteins, hydroxyl groups and carbonyl group reported as major stronger reductant metabolites that used for a bioreduction of metal ions^5,26,77^. Also, the reaction pH value affecting the fabrication of metal oxide nanoparticles; since a natural phytochemicals charge was changed and hence the bioreduction ability was affected, in turn, may affect the shape, size, and yield of metal oxide nanoparticles. There are some metal/metal oxides fabricated at alkaline pH mixture, whereas others prefer very acidic pH mixture values; depends on the nature of phytochemicals and fabricated nanoparticles^8,56^. Temperature is another important factor affecting the biogenic synthesis of metal oxide nanoparticles; because it affects the morphological nanoparticles form and increases the fabrication reaction rate and hence the efficiency of this process. Some reports concluded that high temperatures preferred than room temperature in fabricated some crystal nanoparticles. Furthermore, in some cases, high temperature may destroy the bioreductants that used in nanoparticles reduction process^9,26,54^. So the biogenic synthesis of metal oxide nanoparticles should be developed by using nanobiotechnological strategies to study the nanoparticles reduction parameters. Statistical experimental designs recently used to optimize a nanoparticles biofabrication process. There are few publications studied a nanoparticles biofabrication process and recorded the biosynthesis reaction factors (pH, precursor concentration, temperature, reaction time period and stability of biosynthesized nanoparticles) of the metal oxide nanoparticles (Copper/Copper oxide and Silver nanoparticles) that bio-fabricated by using extracted bioactive metabolites from plant, algae and endophytic fungi^23–25^. In this report, the biosynthesis reaction factors improved through the Taguchi experimental design method approach and the nano-MgO biosynthesis increased about 2.4 times larger than basal condition by using the optimized reaction condition that contained: Metabolites conc. (100%), Mg (NO_3_)_2_·6H_2_O (1 M), Temperature (100 °C) at pH (7.0). Finally, this is the first report using nanobiotechnological strategies to study the nano-MgO (320 g/l) biogenic synthesis parameters by using endophytic *Streptomyces coelicolor* intracellular extract that cultivated in semi industrial scale bioreactor.

The nanoparticle sizes considered as the main factor that affects on the antimicrobial efficiency. The smaller sized MgO nanoparticles have proficient antimicrobial activities has been investigated against both gram positive and gram negative while bigger inhibition sizes were recorded against gram negative only^5,17,59,61,78,79^. There are few reports on the antimicrobial activity of chemical synthetic MgO nanoparticles in the literature to inhibit the growth of human pathogens especially gram positive bacteria than gram negative bacteria^5,78,79^. In this work, nano-MgO (25 nm) applied *in vitro* against multi-drug resident human pathogens (*Shigella flexneri*, *Staphylococcus aureus*, *Streptococcus pneumonia*, and *Klebsiella pneumonia*) and the large inhibition zone recorded against *Shigella flexneri*. Other researchers also reported the minimum inhibitory concentration (MIC) of MgO nanoparticles (>20 nm) was recorded as 0.5, 4 and 8 mg/ml for *S*. *aureus*, *E*. *coli*, *Campylobacter jejuni*, *Staphylococcus epidermidis*, *Bacillus cereus*, *Klebsiella pneumonia*, *Proteus vulgaris* and *Salmonella*^17,59,61,78^. The average of MICs was recorded as 25 µg/ml that inhibited 90% of the pathogenic living cells and compared with 100 mg/ml ampicilin/sulbactam solution that killed 40% of the same pathogenic living cells. Generally, nanoparticles attacked easily microbial cells via their vital components such as peptidoglycan cell wall, cytoplasmic membrane, and bio-molecules to disturb the cell membrane permeability and pore the cytoplasmic membrane so the microbial membranes morphology were severity damaged^78–81^. Subsequently, the nanoparticles reacted with the sulfur-containing proteins as well as a phosphorus-containing compound such as DNA thereby inhibiting protein function and resulting in microbial cell death^61,78,82,83^. Recently, the scientists suggested that the uptake of the nano-MgO may be affected by the cell membrane binding process and internalization process (endocytosis pathway). So the antibacterial efficiency of nanoparticles may associate with the chemical structure of the microbial membranes (gram-positive or gram-negative bacteria)^78,84,85^. Since the nano-MgO may oxidize the lipoproteins on the plasma membrane and disturb the metabolic functions such as respiration resulting in cell decomposition and death eventually^86^. So the using of nano-MgO as an antibacterial agent was active significantly against gram-negative bacteria more than gram-positive bacteria^17,59,84^. Until now, the nano-toxicologists have concluded that the specific mechanisms of the accurate interactions between nanoparticles and pathogenic cell membrane (the first contact point) have been poorly explained^78,82,83^. So the precise bactericidal effect of nanoparticles is not yet clearly detected. Basically, there are many physicochemical parameters of nanoparticles influence their toxicities such as size, shape, surface charge and chemical composition among others^80–86^. Previously, it has been concluded that the highest concentrations of nanoparticles are mainly accumulated as aggregates in the microbial cells without manifesting any acute toxic effects^80^. So the aggregation states of nanoparticles have undesirable effects than the well-dispersed nanoparticles^81,82^. Some reports revealed that the bactericidal effect of nanoparticles depended on the dispersion of the smallest nanoparticles because they can penetrate easily into the pathogen cells, affecting the DNA and the enzymes leading to cell death, but nanoparticles at aggregations statue accumulated on the cell surface and compromise cellular permeability^83,85,86^. Finally, in this work, the low nano-MgO concentration has the lowest affinity for aggregation so its toxicity increased. In this respect, the reported data revealed that smaller nano-MgO particles at low concentration are found to have the greatest antibacterial activity that provided stronger bactericidal interactions, especially in gram-negative strains. The results of this report are expected to gather sufficient knowledge to discover and develop a new simple, cheap and eco-friendly nano-MgO as an extremely strong antimicrobial agent used in biomedical applications.
